# Supplementary material for: Socioeconomic inequalities in health among elderly people in Taiwan: A life course perspective
Source: PLoS One. 2025 Jul 3;20(7):e0327191. doi: 10.1371/journal.pone.0327191 (PMC12225796; doi:10.1371/journal.pone.0327191)
Supplement: S1 File — (DOCX) [file pone.0327191.s001.docx]

**Sensitivity analyses**

1. **Individuals “lost to follow-up” are excluded**

Table 1A

GEE analysis of Self-rated health (SRH): Odds ratios (95% confidence intervals) across three life stages’ socioeconomic status (SES) strata

| Father’s education | Education | Monthly household income | | | | |
| --- | --- | --- | --- | --- | --- | --- |
|  |  | low |  | medium |  | high |
| **Model 1: crude model** | |  |  |  |  |  |
| illiterate | illiterate | 1.00 |  | 1.31*(1.05-1.63) |  | 1.51*(1.01-2.26) |
|  | primary education | 1.47*(1.05-2.07) |  | 2.00***(1.60-2.50) |  | 2.52***(1.83-3.48) |
|  | more than primary | 3.57**(1.59-8.02) |  | 2.86***(1.96-4.16) |  | 3.54***(2.20-5.70) |
| literate | Illiterate | 1.34(0.93-1.95) |  | 1.44*(1.09-1.91) |  | 2.43*(1.23-4.82) |
|  | Primary education | 1.51*(1.02-2.23) |  | 1.94***(1.55-2.42) |  | 2.71***(2.05-3.57) |
|  | more than primary | 2.38**(1.24-4.58) |  | 3.29***(2.59-4.19) |  | 4.27***(3.35-5.44) |
| **Model 2: adjusted for gender and age** | |  |  |  |  |  |
| illiterate | illiterate | 1.00 |  | 1.22(0.98-1.52) |  | 1.39(0.94-2.06) |
|  | primary education | 1.18(0.84-1.66) |  | 1.50***(1.20-1.89) |  | 1.80***(1.30-2.49) |
|  | more than primary | 2.81**(1.28-6.14) |  | 2.03***(1.39-2.98) |  | 2.38***(1.47-3.85) |
| literate | illiterate | 1.45(0.99-2.10) |  | 1.45**(1.10-1.92) |  | 2.34*(1.18-4.62) |
|  | primary education | 1.26(0.86-1.85) |  | 1.54***(1.23-1.93) |  | 2.10***(1.58-2.79) |
|  | more than primary | 1.86(0.92-3.76) |  | 2.44***(1.90-3.12) |  | 3.01***(2.35-3.87) |
| **Model 3: adjusted for gender, age and baseline health** | | |  |  |  |  |
| illiterate | illiterate | 1.00 |  | 1.15(0.95-1.39) |  | 1.21(0.84-1.75) |
|  | primary education | 1.16(0.88-1.55) |  | 1.24*(1.01-1.52) |  | 1.76***(1.27-2.44) |
|  | more than primary | 1.29(0.79-2.12) |  | 1.85**(1.26-2.70) |  | 1.27(0.79-2.06) |
| literate | illiterate | 1.50*(1.04-2.15) |  | 1.48**(1.15-1.91) |  | 2.21**(1.24-3.93) |
|  | primary education | 1.49*(1.02-2.16) |  | 1.41***(1.15-1.73) |  | 1.66***(1.27-2.16) |
|  | more than primary | 1.85**(1.19-2.87) |  | 1.97***(1.56-2.48) |  | 2.13***(1.69-2.68) |

* p<0.05, ** p<0.01, *** p<0.001

Table 1B

GEE analysis of ADL disability (bathing only): Odds ratios (95% confidence intervals) across three life stages’ SES strata

| Father’s education | Education | Monthly household income | | | | |
| --- | --- | --- | --- | --- | --- | --- |
|  |  | low |  | medium |  | high |
| **Model 1: crude model** | |  |  |  |  |  |
| illiterate | illiterate | 1.00 |  | 1.73***(1.32-2.29) |  | 1.57(0.90-2.74) |
|  | primary education | 1.44(0.91-2.29) |  | 1.91***(1.42-2.57) |  | 3.46**(1.63-7.36) |
|  | more than primary | 2.48(0.57-10.78) |  | 1.72(0.95-3.10) |  | 16.00**(2.29-111.84) |
| literate | illiterate | 1.22(0.73-2.03) |  | 1.87**(1.29-2.70) |  | 1.46(0.66-3.26) |
|  | primary education | 1.16(0.69-1.94) |  | 2.03***(1.50-2.74) |  | 3.76***(2.15-6.59) |
|  | more than primary | 1.28(0.47-3.46) |  | 3.69***(2.42-5.62) |  | 3.38***(2.24-5.10) |
| **Model 2: adjusted for gender and age** | |  |  |  |  |  |
| illiterate | illiterate | 1.00 |  | 1.37*(1.03-1.82) |  | 1.07(0.62-1.84) |
|  | primary education | 1.11(0.70-1.76) |  | 1.07(0.78-1.47) |  | 1.69(0.78-3.68) |
|  | more than primary | 1.77(0.42-7.45) |  | 0.94(0.53-1.66) |  | 6.82(0.94-49.18) |
| literate | illiterate | 1.30(0.75-2.25) |  | 1.55*(1.07-2.26) |  | 1.06(0.44-2.53) |
|  | primary education | 0.89(0.53-1.50) |  | 1.22(0.89-1.67) |  | 2.02*(1.15-3.57) |
|  | more than primary | 0.82(0.32-2.13) |  | 2.11***(1.37-3.27) |  | 1.65*(1.07-2.54) |
| **Model 3: adjusted for gender, age and baseline health** | | |  |  |  |  |
| illiterate | illiterate | 1.00 |  | 1.09(0.81-1.46) |  | 0.93(0.52-1.70) |
|  | primary education | 1.25(0.74-2.10) |  | 0.87(0.62-1.21) |  | 2.15(1.00-4.60) |
|  | more than primary | 1.84(0.43-7.83) |  | 0.91(0.45-1.84) |  | 4.16(0.58-30.09) |
| literate | illiterate | 1.09(0.62-1.92) |  | 1.17(0.80-1.71) |  | 0.79(0.33-1.90) |
|  | primary education | 0.69(0.41-1.16) |  | 0.92(0.66-1.29) |  | 1.77(0.93-3.37) |
|  | more than primary | 0.67(0.26-1.74) |  | 1.71*(1.10-2.67) |  | 1.18(0.76-1.83) |

* p<0.05, ** p<0.01, *** p<0.001

Table 1C

GEE analysis of IADL disability: Odds ratios (95% confidence intervals) across three life stages’ SES strata

| Father’s education | Education | Monthly household income | | | | |
| --- | --- | --- | --- | --- | --- | --- |
|  |  | low |  | medium |  | high |
| **Model 1: crude model** | |  |  |  |  |  |
| illiterate | illiterate | 1.00 |  | 2.11***(1.72-2.60) |  | 2.28***(1.55-3.33) |
|  | primary education | 3.63***(2.57-5.15) |  | 7.25***(5.77-9.11) |  | 10.37***(6.83-15.75) |
|  | more than primary | 3.28*(1.27-8.44) |  | 8.93***(5.73-13.91) |  | 33.37***(13.17-84.54) |
| literate | illiterate | 1.33(0.89-1.99) |  | 2.58***(1.97-3.38) |  | 3.08***(1.68-5.66) |
|  | primary education | 3.62***(2.42-5.42) |  | 7.20***(5.73-9.05) |  | 10.38***(7.43-14.51) |
|  | more than primary | 7.05***(2.86-17.39) |  | 12.86***(9.65-17.12) |  | 18.81***(13.68-25.87) |
| **Model 2: adjusted for gender and age** | |  |  |  |  |  |
| illiterate | illiterate | 1.00 |  | 1.59***(1.29-1.96) |  | 1.40*(1.00-1.96) |
|  | primary education | 2.36***(1.65-3.38) |  | 3.36***(2.65-4.27) |  | 3.80***(2.40-6.00) |
|  | more than primary | 1.77(0.70-4.46) |  | 3.48***(2.21-5.46) |  | 10.03***(4.06-24.79) |
| literate | illiterate | 1.54(0.99-2.37) |  | 2.26***(1.74-2.95) |  | 2.18**(1.23-3.85) |
|  | primary education | 2.54***(1.67-3.87) |  | 3.81***(3.02-4.81) |  | 5.04***(3.56-7.12) |
|  | more than primary | 4.08**(1.43-11.65) |  | 6.40***(4.72-8.69) |  | 7.56***(5.50-10.40) |
| **Model 3: adjusted for gender, age and baseline health** | | |  |  |  |  |
| illiterate | illiterate | 1.00 |  | 1.19(0.95-1.49) |  | 1.17(0.71-1.93) |
|  | primary education | 1.39*(1.01-1.92) |  | 1.65***(1.28-2.14) |  | 1.76**(1.16-2.65) |
|  | more than primary | 1.31(0.62-2.75) |  | 1.59*(1.05-2.42) |  | 4.25**(1.69-10.72) |
| literate | illiterate | 1.24(0.79-1.94) |  | 1.44*(1.09-1.92) |  | 1.37(0.63-2.96) |
|  | primary education | 1.78**(1.19-2.66) |  | 1.74***(1.36-2.23) |  | 1.95***(1.37-2.78) |
|  | more than primary | 2.24*(1.11-4.52) |  | 2.75***(2.03-3.73) |  | 3.13***(2.27-4.32) |

* p<0.05, ** p<0.01, *** p<0.001

1. **10% of individuals “lost to follow-up” classified as unhealthy**

Table 2A

GEE analysis of Self-rated health (SRH): Odds ratios (95% confidence intervals) across three life stages’ SES strata

| Father’s education | Education | Monthly household income | | | | |
| --- | --- | --- | --- | --- | --- | --- |
|  |  | low |  | medium |  | high |
| **Model 1: crude model** | |  |  |  |  |  |
| illiterate | illiterate | 1.00 |  | 0.96(0.82-1.12) |  | 1.01(0.75-1.35) |
|  | primary education | 1.07(0.84-1.37) |  | 1.17(0.99-1.38) |  | 1.29(0.98-1.70) |
|  | more than primary | 1.60(0.75-3.43) |  | 1.50**(1.11-2.03) |  | 1.50(0.98-2.28) |
| literate | Illiterate | 1.08(0.81-1.44) |  | 1.05(0.85-1.29) |  | 1.21(0.70-2.09) |
|  | Primary education | 1.02(0.75-1.37) |  | 1.08(0.92-1.28) |  | 1.27*(1.00-1.61) |
|  | more than primary | 1.19(0.64-2.18) |  | 1.69***(1.40-2.04) |  | 1.87***(1.54-2.26) |
| **Model 2: adjusted for gender and age** | |  |  |  |  |  |
| illiterate | illiterate | 1.00 |  | 1.01(0.86-1.18) |  | 1.17(0.87-1.58) |
|  | primary education | 0.91(0.71-1.18) |  | 1.09(0.92-1.30) |  | 1.22(0.93-1.61) |
|  | more than primary | 1.25(0.62-2.52) |  | 1.38(1.00-1.90) |  | 1.40(0.92-2.13) |
| literate | illiterate | 1.15(0.86-1.53) |  | 1.23(1.00-1.52) |  | 1.37(0.78-2.40) |
|  | primary education | 0.94(0.69-1.29) |  | 1.08(0.91-1.28) |  | 1.28*(1.01-1.63) |
|  | more than primary | 1.12(0.60-2.09) |  | 1.60***(1.31-1.95) |  | 1.80***(1.47-2.20) |
| **Model 3: adjusted for gender, age and baseline health** | | |  |  |  |  |
| illiterate | illiterate | 1.00 |  | 0.97(0.82-1.13) |  | 1.12(0.83-1.52) |
|  | primary education | 0.95(0.74-1.21) |  | 0.96(0.80-1.14) |  | 1.12(0.86-1.45) |
|  | more than primary | 0.75(0.41-1.39) |  | 1.23(0.90-1.67) |  | 0.95(0.63-1.45) |
| literate | illiterate | 1.11(0.83-1.48) |  | 1.19(0.97-1.47) |  | 1.26(0.78-2.04) |
|  | primary education | 0.97(0.71-1.33) |  | 0.99(0.83-1.17) |  | 1.01(0.81-1.26) |
|  | more than primary | 0.98(0.62-1.56) |  | 1.33**(1.09-1.61) |  | 1.36**(1.12-1.66) |

* p<0.05, ** p<0.01, *** p<0.001

Table 2B

GEE analysis of ADL disability (bathing only): Odds ratios (95% confidence intervals) across three life stages’ SES strata

| Father’s education | Education | Monthly household income | | | | |
| --- | --- | --- | --- | --- | --- | --- |
|  |  | low |  | medium |  | high |
| **Model 1: crude model** | |  |  |  |  |  |
| illiterate | illiterate | 1.00 |  | 1.40**(1.09-1.81) |  | 1.31(0.78-2.17) |
|  | primary education | 1.02(0.67-1.56) |  | 1.49**(1.14-1.96) |  | 2.49**(1.34-4.62) |
|  | more than primary | 1.31(0.36-4.79) |  | 1.71(0.97-3.03) |  | 12.36*(1.75-87.15) |
| literate | illiterate | 1.19(0.74-1.92) |  | 1.53*(1.08-2.17) |  | 1.21(0.48-3.08) |
|  | primary education | 0.84(0.52-1.36) |  | 1.49**(1.14-1.95) |  | 2.98***(1.81-4.92) |
|  | more than primary | 1.37(0.52-3.58) |  | 2.15***(1.49-3.09) |  | 2.43***(1.67-3.52) |
| **Model 2: adjusted for gender and age** | |  |  |  |  |  |
| illiterate | illiterate | 1.00 |  | 1.20(0.92-1.55) |  | 0.99(0.60-1.65) |
|  | primary education | 0.86(0.56-1.32) |  | 1.01(0.76-1.35) |  | 1.56(0.83-2.94) |
|  | more than primary | 1.06(0.27-4.14) |  | 1.14(0.64-2.01) |  | 6.91(0.96-49.90) |
| literate | illiterate | 1.24(0.76-2.02) |  | 1.34(0.94-1.91) |  | 1.01(0.39-2.64) |
|  | primary education | 0.70(0.44-1.14) |  | 1.04(0.78-1.38) |  | 1.93*(1.16-3.21) |
|  | more than primary | 1.01(0.41-2.49) |  | 1.50*(1.03-2.18) |  | 1.49*(1.01-2.19) |
| **Model 3: adjusted for gender, age and baseline health** | | |  |  |  |  |
| illiterate | illiterate | 1.00 |  | 1.06(0.83-1.37) |  | 0.99(0.58-1.69) |
|  | primary education | 0.94(0.61-1.43) |  | 0.94(0.70-1.24) |  | 1.44(0.79-2.62) |
|  | more than primary | 1.08(0.23-4.93) |  | 1.12(0.65-1.93) |  | 6.74(0.94-48.23) |
| literate | illiterate | 1.19(0.72-1.96) |  | 1.16(0.82-1.62) |  | 0.88(0.40-1.93) |
|  | primary education | 0.63(0.40-1.01) |  | 0.91(0.68-1.20) |  | 1.77*(1.08-2.92) |
|  | more than primary | 1.01(0.48-2.10) |  | 1.40(0.96-2.04) |  | 1.29(0.88-1.91) |

* p<0.05, ** p<0.01, *** p<0.001

Table 2C

GEE analysis of IADL disability: Odds ratios (95% confidence intervals) across three life stages’ SES strata

| Father’s education | Education | Monthly household income | | | | |
| --- | --- | --- | --- | --- | --- | --- |
|  |  | low |  | medium |  | high |
| **Model 1: crude model** | |  |  |  |  |  |
| illiterate | illiterate | 1.00 |  | 1.40***(1.20-1.65) |  | 1.45*(1.04-2.01) |
|  | primary education | 2.07***(1.53-2.80) |  | 3.67***(3.04-4.44) |  | 4.90***(3.28-7.33) |
|  | more than primary | 1.93(0.85-4.38) |  | 4.76***(3.10-7.30) |  | 16.67***(6.87-40.44) |
| literate | illiterate | 1.11(0.81-1.52) |  | 1.70***(1.36-2.12) |  | 1.72(1.00-2.95) |
|  | primary education | 2.03***(1.44-2.88) |  | 3.54***(2.93-4.27) |  | 5.26***(3.88-7.13) |
|  | more than primary | 4.05**(1.75-9.39) |  | 5.99***(4.64-7.74) |  | 8.48***(6.38-11.28) |
| **Model 2: adjusted for gender and age** | |  |  |  |  |  |
| illiterate | illiterate | 1.00 |  | 1.22*(1.04-1.42) |  | 1.15(0.86-1.53) |
|  | primary education | 1.45*(1.09-1.94) |  | 2.16***(1.78-2.63) |  | 2.76***(1.84-4.14) |
|  | more than primary | 1.22(0.52-2.91) |  | 2.63***(1.74-3.98) |  | 8.69***(3.42-22.07) |
| literate | illiterate | 1.24(0.91-1.70) |  | 1.63***(1.32-2.03) |  | 1.61*(1.00-2.59) |
|  | primary education | 1.53*(1.10-2.13) |  | 2.35***(1.94-2.83) |  | 3.22***(2.38-4.35) |
|  | more than primary | 2.55*(1.07-6.12) |  | 3.42***(2.64-4.42) |  | 4.41***(3.34-5.84) |
| **Model 3: adjusted for gender, age and baseline health** | | |  |  |  |  |
| illiterate | illiterate | 1.00 |  | 0.98(0.84-1.16) |  | 1.02(0.72-1.44) |
|  | primary education | 1.02(0.77-1.35) |  | 1.33**(1.09-1.62) |  | 1.53*(1.06-2.22) |
|  | more than primary | 1.08(0.51-2.27) |  | 1.52*(1.00-2.31) |  | 4.52***(1.90-10.72) |
| literate | illiterate | 1.00(0.72-1.37) |  | 1.19(0.97-1.48) |  | 1.19(0.71-2.00) |
|  | primary education | 1.16(0.83-1.62) |  | 1.34**(1.11-1.63) |  | 1.69***(1.25-2.28) |
|  | more than primary | 1.58(0.81-3.07) |  | 1.97***(1.52-2.56) |  | 2.42***(1.82-3.22) |

* p<0.05, ** p<0.01, *** p<0.001

1. **30% of individuals “lost to follow-up” classified as unhealthy**

Table 3A

GEE analysis of Self-rated health (SRH): Odds ratios (95% confidence intervals) across three life stages’ SES strata

| Father’s education | Education | Monthly household income | | | | |
| --- | --- | --- | --- | --- | --- | --- |
|  |  | low |  | medium |  | high |
| **Model 1: crude model** | |  |  |  |  |  |
| illiterate | illiterate | 1.00 |  | 1.04(0.88-1.22) |  | 1.18(0.87-1.59) |
|  | primary education | 0.99(0.76-1.28) |  | 1.33***(1.13-1.58) |  | 1.53**(1.18-1.99) |
|  | more than primary | 1.20(0.56-2.56) |  | 1.54**(1.13-2.11) |  | 2.03**(1.33-3.09) |
| literate | Illiterate | 1.08(0.81-1.43) |  | 1.15(0.94-1.42) |  | 1.20(0.68-2.10) |
|  | Primary education | 1.12(0.81-1.54) |  | 1.21*(1.02-1.44) |  | 1.42**(1.12-1.80) |
|  | more than primary | 1.36(0.76-2.42) |  | 1.91***(1.58-2.32) |  | 2.17***(1.78-2.64) |
| **Model 2: adjusted for gender and age** | |  |  |  |  |  |
| illiterate | illiterate | 1.00 |  | 1.06(0.90-1.24) |  | 1.24(0.92-1.67) |
|  | primary education | 0.84(0.65-1.10) |  | 1.20*(1.01-1.43) |  | 1.38*(1.06-1.80) |
|  | more than primary | 0.99(0.46-2.17) |  | 1.32(0.96-1.82) |  | 1.82**(1.19-2.78) |
| literate | illiterate | 1.14(0.86-1.50) |  | 1.24*(1.01-1.53) |  | 1.28(0.72-2.26) |
|  | primary education | 1.00(0.73-1.39) |  | 1.14(0.96-1.36) |  | 1.36*(1.07-1.72) |
|  | more than primary | 1.23(0.67-2.27) |  | 1.69***(1.38-2.06) |  | 1.94***(1.58-2.38) |
| **Model 3: adjusted for gender, age and baseline health** | | |  |  |  |  |
| illiterate | illiterate | 1.00 |  | 1.00(0.85-1.17) |  | 1.23(0.90-1.68) |
|  | primary education | 0.87(0.67-1.11) |  | 1.03(0.86-1.22) |  | 1.25(0.96-1.63) |
|  | more than primary | 0.53(0.27-1.01) |  | 1.17(0.86-1.60) |  | 1.20(0.78-1.84) |
| literate | illiterate | 1.12(0.84-1.49) |  | 1.24*(1.01-1.53) |  | 1.17(0.72-1.89) |
|  | primary education | 1.05(0.76-1.44) |  | 1.03(0.86-1.22) |  | 1.04(0.83-1.30) |
|  | more than primary | 1.08(0.71-1.65) |  | 1.42***(1.16-1.73) |  | 1.46***(1.19-1.78) |

* p<0.05, ** p<0.01, *** p<0.001

Table 3B

GEE analysis of ADL disability (bathing only): Odds ratios (95% confidence intervals) across three life stages’ SES strata

| Father’s education | Education | Monthly household income | | | | |
| --- | --- | --- | --- | --- | --- | --- |
|  |  | low |  | medium |  | high |
| **Model 1: crude model** | |  |  |  |  |  |
| illiterate | illiterate | 1.00 |  | 1.46***(1.17-1.82) |  | 1.54(0.96-2.49) |
|  | primary education | 0.92(0.64-1.33) |  | 1.62***(1.27-2.06) |  | 2.54***(1.55-4.17) |
|  | more than primary | 0.80(0.26-2.44) |  | 1.47(0.94-2.32) |  | 19.81**(2.81-139.67) |
| literate | illiterate | 1.14(0.75-1.73) |  | 1.55**(1.14-2.11) |  | 1.11(0.54-2.28) |
|  | primary education | 1.05(0.68-1.62) |  | 1.60***(1.25-2.04) |  | 2.33***(1.59-3.42) |
|  | more than primary | 1.47(0.67-3.39) |  | 1.95***(1.44-2.65) |  | 2.46***(1.79-3.39) |
| **Model 2: adjusted for gender and age** | |  |  |  |  |  |
| illiterate | illiterate | 1.00 |  | 1.22(0.97-1.53) |  | 1.15(0.72-1.83) |
|  | primary education | 0.84(0.58-1.21) |  | 1.14(0.88-1.47) |  | 1.69*(1.01-2.80) |
|  | more than primary | 0.69(0.24-1.99) |  | 1.02(0.65-1.62) |  | 12.00*(1.67-86.16) |
| literate | illiterate | 1.16(0.75-1.80) |  | 1.29(0.95-1.76) |  | 0.88(0.45-1.73) |
|  | primary education | 0.93(0.60-1.45) |  | 1.15(0.89-1.48) |  | 1.56*(1.05-2.31) |
|  | more than primary | 1.14(0.54-2.40) |  | 1.43*(1.05-1.96) |  | 1.60**(1.14-2.23) |
| **Model 3: adjusted for gender, age and baseline health** | | |  |  |  |  |
| illiterate | illiterate | 1.00 |  | 1.12(0.90-1.40) |  | 1.15(0.72-1.84) |
|  | primary education | 0.89(0.62-1.27) |  | 1.11(0.87-1.43) |  | 1.65(1.00-2.73) |
|  | more than primary | 0.66(0.23-1.84) |  | 1.04(0.64-1.68) |  | 13.36**(1.88-95.09) |
| literate | illiterate | 1.15(0.75-1.75) |  | 1.18(0.87-1.59) |  | 0.78(0.41-1.49) |
|  | primary education | 0.84(0.54-1.31) |  | 1.04(0.81-1.34) |  | 1.52*(1.02-2.27) |
|  | more than primary | 1.21(0.60-2.43) |  | 1.40*(1.03-1.91) |  | 1.45*(1.03-2.04) |

* p<0.05, ** p<0.01, *** p<0.001

Table 3C

GEE analysis of IADL disability: Odds ratios (95% confidence intervals) across three life stages’ SES strata

| Father’s education | Education | Monthly household income | | | | |
| --- | --- | --- | --- | --- | --- | --- |
|  |  | low |  | medium |  | high |
| **Model 1: crude model** | |  |  |  |  |  |
| illiterate | illiterate | 1.00 |  | 1.53***(1.30-1.79) |  | 1.68**(1.22-2.33) |
|  | primary education | 1.91***(1.43-2.54) |  | 3.89***(3.24-4.68) |  | 5.57***(3.85-8.06) |
|  | more than primary | 1.40(0.55-3.57) |  | 4.29***(2.95-6.23) |  | 24.59***(9.63-62.76) |
| literate | illiterate | 1.13(0.83-1.54) |  | 1.85***(1.49-2.31) |  | 1.72*(1.01-2.93) |
|  | primary education | 2.22***(1.58-3.13) |  | 3.73***(3.11-4.49) |  | 5.09***(3.84-6.74) |
|  | more than primary | 4.27***(2.10-8.68) |  | 5.43***(4.26-6.92) |  | 7.80***(5.98-10.16) |
| **Model 2: adjusted for gender and age** | |  |  |  |  |  |
| illiterate | illiterate | 1.00 |  | 1.26**(1.07-1.48) |  | 1.25(0.93-1.69) |
|  | primary education | 1.36*(1.02-1.81) |  | 2.23***(1.84-2.71) |  | 2.92***(2.00-4.28) |
|  | more than primary | 0.91(0.36-2.30) |  | 2.26***(1.55-3.28) |  | 11.86***(4.65-30.22) |
| literate | illiterate | 1.23(0.89-1.70) |  | 1.65***(1.33-2.05) |  | 1.45(0.93-2.26) |
|  | primary education | 1.68**(1.20-2.37) |  | 2.34***(1.93-2.83) |  | 2.98***(2.25-3.95) |
|  | more than primary | 2.68**(1.27-5.65) |  | 3.15***(2.45-4.05) |  | 4.03***(3.08-5.28) |
| **Model 3: adjusted for gender, age and baseline health** | | |  |  |  |  |
| illiterate | illiterate | 1.00 |  | 1.01(0.85-1.19) |  | 1.10(0.77-1.57) |
|  | primary education | 0.92(0.69-1.21) |  | 1.38**(1.12-1.69) |  | 1.65**(1.16-2.33) |
|  | more than primary | 0.77(0.39-1.51) |  | 1.32(0.89-1.96) |  | 6.28***(2.64-14.96) |
| literate | illiterate | 0.98(0.72-1.34) |  | 1.20(0.97-1.49) |  | 1.04(0.64-1.67) |
|  | primary education | 1.23(0.87-1.73) |  | 1.33**(1.09-1.62) |  | 1.58**(1.18-2.13) |
|  | more than primary | 1.74(0.99-3.06) |  | 1.82***(1.41-2.33) |  | 2.20***(1.67-2.89) |

* p<0.05, ** p<0.01, *** p<0.001

1. **50% of individuals “lost to follow-up” classified as unhealthy**

Table 4A

GEE analysis of Self-rated health (SRH): Odds ratios (95% confidence intervals) across three life stages’ SES strata

| Father’s education | Education | Monthly household income | | | | |
| --- | --- | --- | --- | --- | --- | --- |
|  |  | low |  | medium |  | high |
| **Model 1: crude model** | |  |  |  |  |  |
| illiterate | illiterate | 1.00 |  | 1.21*(1.02-1.44) |  | 1.23(0.90-1.68) |
|  | primary education | 1.22(0.93-1.61) |  | 1.58***(1.32-1.89) |  | 1.90***(1.44-2.51) |
|  | more than primary | 1.64(0.78-3.42) |  | 1.97***(1.43-2.70) |  | 2.40***(1.56-3.68) |
| literate | Illiterate | 1.37*(1.01-1.85) |  | 1.38**(1.09-1.73) |  | 1.56(0.84-2.93) |
|  | Primary education | 1.38*(1.00-1.90) |  | 1.50***(1.25-1.79) |  | 1.85***(1.46-2.35) |
|  | more than primary | 2.02**(1.19-3.41) |  | 2.15***(1.76-2.63) |  | 2.67***(2.18-3.27) |
| **Model 2: adjusted for gender and age** | |  |  |  |  |  |
| illiterate | illiterate | 1.00 |  | 1.18(1.00-1.41) |  | 1.20(0.88-1.64) |
|  | primary education | 1.06(0.81-1.39) |  | 1.35**(1.12-1.63) |  | 1.61**(1.21-2.13) |
|  | more than primary | 1.38(0.66-2.90) |  | 1.62**(1.17-2.25) |  | 1.97**(1.27-3.05) |
| literate | illiterate | 1.45*(1.07-1.96) |  | 1.41**(1.12-1.77) |  | 1.57(0.84-2.94) |
|  | primary education | 1.24(0.90-1.70) |  | 1.33**(1.11-1.60) |  | 1.64***(1.28-2.09) |
|  | more than primary | 1.77*(1.03-3.05) |  | 1.83***(1.48-2.25) |  | 2.23***(1.81-2.76) |
| **Model 3: adjusted for gender, age and baseline health** | | |  |  |  |  |
| illiterate | illiterate | 1.00 |  | 1.14(0.96-1.34) |  | 1.15(0.85-1.55) |
|  | primary education | 1.13(0.88-1.45) |  | 1.18(0.99-1.41) |  | 1.48**(1.14-1.94) |
|  | more than primary | 0.79(0.44-1.45) |  | 1.45*(1.06-1.97) |  | 1.32(0.89-1.95) |
| literate | illiterate | 1.42*(1.05-1.94) |  | 1.38**(1.11-1.72) |  | 1.46(0.87-2.45) |
|  | primary education | 1.30(0.96-1.78) |  | 1.21*(1.01-1.44) |  | 1.27*(1.01-1.60) |
|  | more than primary | 1.59*(1.06-2.40) |  | 1.50***(1.23-1.83) |  | 1.66***(1.36-2.03) |

* p<0.05, ** p<0.01, *** p<0.001

Table 4B

GEE analysis of ADL disability (bathing only): Odds ratios (95% confidence intervals) across three life stages’ SES strata

| Father’s education | Education | Monthly household income | | | | |
| --- | --- | --- | --- | --- | --- | --- |
|  |  | low |  | medium |  | high |
| **Model 1: crude model** | |  |  |  |  |  |
| illiterate | illiterate | 1.00 |  | 1.56***(1.28-1.91) |  | 1.38(0.92-2.08) |
|  | primary education | 1.09(0.79-1.51) |  | 1.79***(1.44-2.23) |  | 2.70***(1.72-4.23) |
|  | more than primary | 1.27(0.44-3.68) |  | 1.73*(1.13-2.64) |  | 5.16***(2.14-12.41) |
| literate | illiterate | 1.32(0.92-1.88) |  | 1.54**(1.18-2.01) |  | 1.55(0.76-3.13) |
|  | primary education | 1.18(0.79-1.78) |  | 1.75***(1.42-2.17) |  | 2.59***(1.81-3.71) |
|  | more than primary | 1.98(0.92-4.28) |  | 1.72***(1.33-2.22) |  | 2.37***(1.81-3.12) |
| **Model 2: adjusted for gender and age** | |  |  |  |  |  |
| illiterate | illiterate | 1.00 |  | 1.29*(1.06-1.58) |  | 0.98(0.65-1.47) |
|  | primary education | 1.04(0.74-1.45) |  | 1.28*(1.01-1.61) |  | 1.79*(1.14-2.83) |
|  | more than primary | 1.16(0.43-3.10) |  | 1.24(0.81-1.90) |  | 3.07*(1.29-7.30) |
| literate | illiterate | 1.35(0.93-1.96) |  | 1.25(0.96-1.64) |  | 1.18(0.62-1.62) |
|  | primary education | 1.08(0.72-1.62) |  | 1.26*(1.01-1.57) |  | 1.72**(1.21-2.44) |
|  | more than primary | 1.59(0.80-3.18) |  | 1.29(0.99-1.69) |  | 1.54**(1.16-2.05) |
| **Model 3: adjusted for gender, age and baseline health** | | |  |  |  |  |
| illiterate | illiterate | 1.00 |  | 1.21(0.99-1.48) |  | 0.96(0.63-1.46) |
|  | primary education | 1.14(0.81-1.61) |  | 1.24(0.99-1.57) |  | 1.75*(1.13-2.71) |
|  | more than primary | 1.15(0.44-3.03) |  | 1.23(0.80-1.88) |  | 3.29**(1.36-8.00) |
| literate | illiterate | 1.39(0.94-2.06) |  | 1.14(0.88-1.50) |  | 1.01(0.54-1.90) |
|  | primary education | 1.01(0.70-1.47) |  | 1.18(0.94-1.48) |  | 1.66**(1.16-2.37) |
|  | more than primary | 1.77(0.84-3.70) |  | 1.23(0.94-1.62) |  | 1.44*(1.08-1.92) |

* p<0.05, ** p<0.01, *** p<0.001

Table 4C

GEE analysis of IADL disability: Odds ratios (95% confidence intervals) across three life stages’ SES strata

| Father’s education | Education | Monthly household income | | | | |
| --- | --- | --- | --- | --- | --- | --- |
|  |  | low |  | medium |  | high |
| **Model 1: crude model** | |  |  |  |  |  |
| illiterate | illiterate | 1.00 |  | 1.75***(1.48-2.07) |  | 1.70**(1.22-2.36) |
|  | primary education | 2.22***(1.66-2.98) |  | 4.32***(3.57-5.22) |  | 6.16***(4.24-8.93) |
|  | more than primary | 2.00(0.78-5.12) |  | 5.01***(3.49-7.21) |  | 14.95***(6.93-32.25) |
| literate | illiterate | 1.35(0.98-1.86) |  | 2.07***(1.66-2.59) |  | 2.24**(1.26-3.98) |
|  | primary education | 2.55***(1.79-3.63) |  | 4.20***(3.48-5.07) |  | 6.03***(4.50-8.08) |
|  | more than primary | 5.53***(2.58-11.83) |  | 5.04***(4.03-6.32) |  | 7.73***(6.01-9.92) |
| **Model 2: adjusted for gender and age** | |  |  |  |  |  |
| illiterate | illiterate | 1.00 |  | 1.41***(1.18-1.67) |  | 1.18(0.86-1.61) |
|  | primary education | 1.68***(1.25-2.25) |  | 2.47***(2.02-3.01) |  | 3.20***(2.19-4.68) |
|  | more than primary | 1.39(0.57-3.40) |  | 2.69***(1.87-3.86) |  | 6.99***(3.25-15.04) |
| literate | illiterate | 1.48*(1.06-2.07) |  | 1.79***(1.43-2.23) |  | 1.81*(1.12-2.94) |
|  | primary education | 2.00***(1.42-2.82) |  | 2.60***(2.14-3.15) |  | 3.43***(2.58-4.57) |
|  | more than primary | 3.62**(1.67-7.85) |  | 2.97***(2.34-3.76) |  | 3.95***(3.07-5.09) |
| **Model 3: adjusted for gender, age and baseline health** | | |  |  |  |  |
| illiterate | illiterate | 1.00 |  | 1.13(0.95-1.34) |  | 0.99(0.69-1.42) |
|  | primary education | 1.15(0.87-1.53) |  | 1.49***(1.22-1.82) |  | 1.77**(1.25-2.48) |
|  | more than primary | 1.21(0.62-2.36) |  | 1.55*(1.08-2.24) |  | 4.07***(2.00-8.31) |
| literate | illiterate | 1.22(0.86-1.72) |  | 1.29*(1.03-1.61) |  | 1.25(0.73-2.15) |
|  | primary education | 1.49*(1.09-2.05) |  | 1.49***(1.22-1.81) |  | 1.82***(1.36-2.43) |
|  | more than primary | 2.44**(1.42-4.18) |  | 1.67***(1.32-2.11) |  | 2.17***(1.68-2.80) |

* p<0.05, ** p<0.01, *** p<0.001

1. **70% of individuals “lost to follow-up” classified as unhealthy**

Table 5A

GEE analysis of Self-rated health (SRH): Odds ratios (95% confidence intervals) across three life stages’ SES strata

| Father’s education | Education | Monthly household income | | | | |
| --- | --- | --- | --- | --- | --- | --- |
|  |  | low |  | medium |  | high |
| **Model 1: crude model** | |  |  |  |  |  |
| illiterate | illiterate | 1.00 |  | 1.18(0.98-1.41) |  | 1.43*(1.02-2.00) |
|  | primary education | 1.17(0.88-1.56) |  | 1.69***(1.41-2.03) |  | 2.09***(1.58-2.78) |
|  | more than primary | 1.65(0.82-3.30) |  | 2.05***(1.50-2.81) |  | 2.81***(1.74-4.53) |
| literate | Illiterate | 1.12(0.83-1.53) |  | 1.37**(1.08-1.73) |  | 1.77(0.99-3.17) |
|  | Primary education | 1.40(0.99-1.99) |  | 1.62***(1.35-1.95) |  | 2.15***(1.68-2.75) |
|  | more than primary | 1.94*(1.11-3.40) |  | 2.26***(1.83-2.79) |  | 3.09***(2.51-3.81) |
| **Model 2: adjusted for gender and age** | |  |  |  |  |  |
| illiterate | illiterate | 1.00 |  | 1.11(0.92-1.33) |  | 1.31(0.93-1.85) |
|  | primary education | 0.99(0.74-1.31) |  | 1.35**(1.11-1.63) |  | 1.62***(1.22-2.16) |
|  | more than primary | 1.34(0.68-2.66) |  | 1.57**(1.14-2.17) |  | 2.10**(1.29-3.41) |
| literate | illiterate | 1.18(0.87-1.62) |  | 1.35*(1.06-1.71) |  | 1.70(0.96-3.02) |
|  | primary education | 1.22(0.87-1.73) |  | 1.34**(1.11-1.63) |  | 1.75***(1.37-2.25) |
|  | more than primary | 1.59(0.89-2.85) |  | 1.80***(1.44-2.24) |  | 2.37***(1.91-2.95) |
| **Model 3: adjusted for gender, age and baseline health** | | |  |  |  |  |
| illiterate | illiterate | 1.00 |  | 1.05(0.88-1.24) |  | 1.25(0.90-1.75) |
|  | primary education | 1.05(0.81-1.36) |  | 1.15(0.96-1.38) |  | 1.49**(1.13-1.96) |
|  | more than primary | 0.72(0.41-1.25) |  | 1.38*(1.01-1.88) |  | 1.35(0.84-2.16) |
| literate | illiterate | 1.14(0.85-1.53) |  | 1.30*(1.03-1.64) |  | 1.58(0.99-2.51) |
|  | primary education | 1.30(0.94-1.79) |  | 1.21*(1.01-1.45) |  | 1.33*(1.05-1.68) |
|  | more than primary | 1.40(0.98-2.00) |  | 1.43***(1.17-1.76) |  | 1.72***(1.40-2.11) |

* p<0.05, ** p<0.01, *** p<0.001

Table 5B

GEE analysis of ADL disability (bathing only): Odds ratios (95% confidence intervals) across three life stages’ SES strata

| Father’s education | Education | Monthly household income | | | | |
| --- | --- | --- | --- | --- | --- | --- |
|  |  | low |  | medium |  | high |
| **Model 1: crude model** | |  |  |  |  |  |
| illiterate | illiterate | 1.00 |  | 1.43***(1.19-1.71) |  | 1.52*(1.04-2.22) |
|  | primary education | 0.97(0.73-1.29) |  | 1.69***(1.39-2.05) |  | 2.46***(1.68-3.60) |
|  | more than primary | 1.11(0.45-2.74) |  | 1.61*(1.11-2.33) |  | 4.20***(2.00-8.82) |
| literate | illiterate | 1.04(0.76-1.42) |  | 1.53***(1.20-1.97) |  | 1.48(0.83-2.64) |
|  | primary education | 1.29(0.88-1.90) |  | 1.73***(1.42-2.11) |  | 2.70***(1.95-3.74) |
|  | more than primary | 1.90(0.97-3.70) |  | 1.67***(1.33-2.09) |  | 2.37***(1.86-3.04) |
| **Model 2: adjusted for gender and age** | |  |  |  |  |  |
| illiterate | illiterate | 1.00 |  | 1.17(0.98-1.41) |  | 1.08(0.75-1.56) |
|  | primary education | 0.93(0.70-1.24) |  | 1.22(0.99-1.50) |  | 1.66**(1.14-2.41) |
|  | more than primary | 1.02(0.45-2.30) |  | 1.17(0.81-1.70) |  | 2.56**(1.27-5.15) |
| literate | illiterate | 1.04(0.76-1.43) |  | 1.24(0.96-1.60) |  | 1.13(0.67-1.92) |
|  | primary education | 1.19(0.81-1.75) |  | 1.25*(1.02-1.54) |  | 1.82***(1.30-2.53) |
|  | more than primary | 1.53(0.80-2.93) |  | 1.26(1.00-1.60) |  | 1.57***(1.21-2.04) |
| **Model 3: adjusted for gender, age and baseline health** | | |  |  |  |  |
| illiterate | illiterate | 1.00 |  | 1.09(0.91-1.30) |  | 1.06(0.70-1.58) |
|  | primary education | 0.98(0.73-1.32) |  | 1.17(0.95-1.44) |  | 1.62*(1.10-2.40) |
|  | more than primary | 0.97(0.42-2.22) |  | 1.18(0.81-1.71) |  | 2.53*(1.23-5.20) |
| literate | illiterate | 1.05(0.77-1.44) |  | 1.09(0.85-1.41) |  | 1.01(0.60-1.71) |
|  | primary education | 1.05(0.73-1.51) |  | 1.15(0.94-1.41) |  | 1.75**(1.23-2.49) |
|  | more than primary | 1.68(0.78-3.64) |  | 1.18(0.92-1.50) |  | 1.44**(1.10-1.88) |

* p<0.05, ** p<0.01, *** p<0.001

Table 5C

GEE analysis of IADL disability: Odds ratios (95% confidence intervals) across three life stages’ SES strata

| Father’s education | Education | Monthly household income | | | | |
| --- | --- | --- | --- | --- | --- | --- |
|  |  | low |  | medium |  | high |
| **Model 1: crude model** | |  |  |  |  |  |
| illiterate | illiterate | 1.00 |  | 1.75***(1.47-2.08) |  | 1.95***(1.38-2.74) |
|  | primary education | 2.12***(1.60-2.80) |  | 4.36***(3.63-5.25) |  | 6.19***(4.40-8.71) |
|  | more than primary | 1.95(0.74-5.14) |  | 4.83***(3.37-6.93) |  | 14.00***(6.23-31.43) |
| literate | illiterate | 1.14(0.81-1.60) |  | 2.13***(1.70-2.68) |  | 2.39**(1.40-4.09) |
|  | primary education | 2.72***(1.89-3.91) |  | 4.39***(3.64-5.29) |  | 6.53***(4.91-8.69) |
|  | more than primary | 5.26***(2.60-10.64) |  | 4.96***(3.99-6.17 |  | 7.99***(6.31-10.13) |
| **Model 2: adjusted for gender and age** | |  |  |  |  |  |
| illiterate | illiterate | 1.00 |  | 1.37***(1.15-1.62) |  | 1.31(0.94-1.82) |
|  | primary education | 1.60**(1.20-2.12) |  | 2.44***(2.01-2.96) |  | 3.14***(2.24-4.41) |
|  | more than primary | 1.34(0.53-3.38) |  | 2.53***(1.76-3.63) |  | 6.39***(2.99-13.67) |
| literate | illiterate | 1.23(0.87-1.72) |  | 1.79***(1.43-2.25) |  | 1.88**(1.22-2.91) |
|  | primary education | 2.13***(1.49-3.07) |  | 2.65***(2.19-3.21) |  | 3.64***(2.74-4.83) |
|  | more than primary | 3.40**(1.59-7.31) |  | 2.87***(2.29-3.61) |  | 4.01***(3.16-5.09) |
| **Model 3: adjusted for gender, age and baseline health** | | |  |  |  |  |
| illiterate | illiterate | 1.00 |  | 1.05(0.88-1.25) |  | 1.08(0.72-1.62) |
|  | primary education | 1.05(0.80-1.37) |  | 1.41***(1.16-1.72) |  | 1.66**(1.20-2.30) |
|  | more than primary | 1.13(0.56-2.29) |  | 1.41*(1.01-1.98) |  | 3.60***(1.74-7.43) |
| literate | illiterate | 0.96(0.70-1.33) |  | 1.20(0.95-1.51) |  | 1.30(0.76-2.24) |
|  | primary education | 1.48*(1.07-2.04) |  | 1.43***(1.18-1.74) |  | 1.82***(1.36-2.45) |
|  | more than primary | 2.18**(1.22-3.89) |  | 1.51***(1.20-1.90) |  | 2.08***(1.62-2.67) |

* p<0.05, ** p<0.01, *** p<0.001

1. **80% of individuals “lost to follow-up” classified as unhealthy**

Table 6A

GEE analysis of Self-rated health (SRH): Odds ratios (95% confidence intervals) across three life stages’ SES strata

| Father’s education | Education | Monthly household income | | | | |
| --- | --- | --- | --- | --- | --- | --- |
|  |  | low |  | medium |  | high |
| **Model 1: crude model** | |  |  |  |  |  |
| illiterate | illiterate | 1.00 |  | 1.26*(1.05-1.51) |  | 1.29(0.89-1.86) |
|  | primary education | 1.31(0.97-1.78) |  | 1.75***(1.44-2.11) |  | 2.19***(1.65-2.91) |
|  | more than primary | 1.92(0.95-3.89) |  | 2.53***(1.80-3.56) |  | 3.05***(1.93-4.84) |
| literate | Illiterate | 1.11(0.79-1.55) |  | 1.49**(1.17-1.89) |  | 2.08*(1.09-3.97) |
|  | Primary education | 1.35(0.97-1.88) |  | 1.77***(1.47-2.14) |  | 2.39***(1.88-3.03) |
|  | more than primary | 1.75(0.96-3.18) |  | 2.45***(1.99-3.01) |  | 3.42***(2.76-4.23) |
| **Model 2: adjusted for gender and age** | |  |  |  |  |  |
| illiterate | illiterate | 1.00 |  | 1.14(0.95-1.37) |  | 1.12(0.78-1.61) |
|  | primary education | 1.07(0.80-1.45) |  | 1.30**(1.07-1.58) |  | 1.57**(1.18-2.09) |
|  | more than primary | 1.51(0.76-3.01) |  | 1.80***(1.28-2.54) |  | 2.10**(1.33-3.31) |
| literate | illiterate | 1.18(0.84-1.66) |  | 1.42**(1.11-1.81) |  | 1.92*(1.02-3.62) |
|  | primary education | 1.15(0.83-1.58) |  | 1.38**(1.13-1.68) |  | 1.82***(1.43-2.32) |
|  | more than primary | 1.34(0.71-2.54) |  | 1.83***(1.48-2.26) |  | 2.43***(1.94-3.03) |
| **Model 3: adjusted for gender, age and baseline health** | | |  |  |  |  |
| illiterate | illiterate | 1.00 |  | 1.07(0.90-1.27) |  | 1.06(0.77-1.45) |
|  | primary education | 1.16(0.90-1.49) |  | 1.08(0.90-1.29) |  | 1.40*(1.06-1.86) |
|  | more than primary | 0.76(0.42-1.35) |  | 1.60**(1.17-2.21) |  | 1.28(0.85-1.93) |
| literate | illiterate | 1.15(0.84-1.57) |  | 1.41**(1.12-1.77) |  | 1.82*(1.06-3.12) |
|  | primary education | 1.24(0.92-1.66) |  | 1.23*(1.03-1.48) |  | 1.35*(1.07-1.70) |
|  | more than primary | 1.17(0.70-1.96) |  | 1.44***(1.18-1.75) |  | 1.72***(1.40-2.11) |

* p<0.05, ** p<0.01, *** p<0.001

Table 6B

GEE analysis of ADL disability (bathing only): Odds ratios (95% confidence intervals) across three life stages’ SES strata

| Father’s education | Education | Monthly household income | | | | |
| --- | --- | --- | --- | --- | --- | --- |
|  |  | low |  | medium |  | high |
| **Model 1: crude model** | |  |  |  |  |  |
| illiterate | illiterate | 1.00 |  | 1.42***(1.20-1.69) |  | 1.57*(1.09-2.27) |
|  | primary education | 1.16(0.88-1.53) |  | 1.58***(1.32-1.89) |  | 2.14***(1.52-3.00) |
|  | more than primary | 1.43(0.56-3.67) |  | 1.69**(1.19-2.40) |  | 3.37***(1.73-6.55) |
| literate | illiterate | 1.13(0.82-1.56) |  | 1.34**(1.08-1.67) |  | 1.47(0.82-2.64) |
|  | primary education | 1.27(0.89-1.83) |  | 1.65***(1.37-1.97) |  | 2.25***(1.68-3.02) |
|  | more than primary | 1.73(0.91-3.26) |  | 1.59***(1.28-1.96) |  | 2.35***(1.85-2.97) |
| **Model 2: adjusted for gender and age** | |  |  |  |  |  |
| illiterate | illiterate | 1.00 |  | 1.16(0.98-1.37) |  | 1.09(0.76-1.58) |
|  | primary education | 1.12(0.85-1.47) |  | 1.11(0.92-1.35) |  | 1.40*(1.00-1.96) |
|  | more than primary | 1.32(0.56-3.11) |  | 1.21(0.85-1.72) |  | 1.99*(1.06-3.72) |
| literate | illiterate | 1.14(0.82-1.59) |  | 1.07(0.86-1.33) |  | 1.11(0.66-1.88) |
|  | primary education | 1.17(0.81-1.68) |  | 1.17(0.97-1.41) |  | 1.47**(1.10-1.98) |
|  | more than primary | 1.36(0.76-2.42) |  | 1.17(0.94-1.46) |  | 1.51***(1.18-1.93) |
| **Model 3: adjusted for gender, age and baseline health** | | |  |  |  |  |
| illiterate | illiterate | 1.00 |  | 1.05(0.90-1.24) |  | 1.06(0.73-1.54) |
|  | primary education | 1.17(0.89-1.54) |  | 1.05(0.87-1.25) |  | 1.37(0.98-1.93) |
|  | more than primary | 1.35(0.58-3.13) |  | 1.21(0.86-1.71) |  | 1.73(0.92-3.24) |
| literate | illiterate | 1.11(0.83-1.48) |  | 0.94(0.76-1.16) |  | 1.00(0.59-1.68) |
|  | primary education | 1.13(0.81-1.58) |  | 1.05(0.88-1.25) |  | 1.35*(1.01-1.80) |
|  | more than primary | 1.31(0.67-2.57) |  | 1.06(0.86-1.32) |  | 1.35*(1.07-1.71) |

* p<0.05, ** p<0.01, *** p<0.001

Table 6C

GEE analysis of IADL disability: Odds ratios (95% confidence intervals) across three life stages’ SES strata

| Father’s education | Education | Monthly household income | | | | |
| --- | --- | --- | --- | --- | --- | --- |
|  |  | low |  | medium |  | high |
| **Model 1: crude model** | |  |  |  |  |  |
| illiterate | illiterate | 1.00 |  | 1.69***(1.42-2.02) |  | 1.78***(1.27-2.50) |
|  | primary education | 2.22***(1.67-2.94) |  | 3.95***(3.28-4.76) |  | 5.22***(3.77-7.24) |
|  | more than primary | 2.21(0.86-5.72) |  | 5.31***(3.67-7.69) |  | 19.69***(9.12-42.48) |
| literate | illiterate | 1.09(0.78-1.54) |  | 1.90***(1.52-2.39) |  | 2.76***(1.58-4.83) |
|  | primary education | 2.16***(1.51-3.07) |  | 4.24***(3.51-5.12) |  | 6.17***(4.65-8.20) |
|  | more than primary | 5.45***(2.49-11.92) |  | 4.73***(3.81-5.88) |  | 7.20***(5.69-9.12) |
| **Model 2: adjusted for gender and age** | |  |  |  |  |  |
| illiterate | illiterate | 1.00 |  | 1.29**(1.08-1.54) |  | 1.15(0.85-1.55) |
|  | primary education | 1.68***(1.26-2.24) |  | 2.14***(1.76-2.60) |  | 2.53***(1.83-3.51) |
|  | more than primary | 1.54(0.61-3.84) |  | 2.73***(1.90-3.94) |  | 8.68***(3.92-19.18) |
| literate | illiterate | 1.17(0.82-1.66) |  | 1.57***(1.25-1.96) |  | 2.15**(1.35-3.43) |
|  | primary education | 1.66**(1.16-2.36) |  | 2.50***(2.06-3.02) |  | 3.32***(2.50-4.42) |
|  | more than primary | 3.49**(1.55-7.89) |  | 2.67**(2.13-3.34) |  | 3.47***(2.73-4.41) |
| **Model 3: adjusted for gender, age and baseline health** | | |  |  |  |  |
| illiterate | illiterate | 1.00 |  | 0.94(0.79-1.13) |  | 0.88(0.60-1.29) |
|  | primary education | 1.03(0.78-1.35) |  | 1.14(0.94-1.39) |  | 1.25(0.91-1.72) |
|  | more than primary | 1.20(0.53-2.69) |  | 1.45*(1.02-2.05) |  | 4.50**(1.79-11.31) |
| literate | illiterate | 0.86(0.61-1.21) |  | 1.00(0.79-1.26) |  | 1.41(0.77-2.58) |
|  | primary education | 1.08(0.78-1.50) |  | 1.27*(1.04-1.54) |  | 1.55**(1.16-2.07) |
|  | more than primary | 2.08*(1.12-3.87) |  | 1.30*(1.04-1.63) |  | 1.67***(1.31-2.14) |

* p<0.05, ** p<0.01, *** p<0.001

1. **90% of individuals “lost to follow-up” classified as unhealthy**

Table 7A

GEE analysis of Self-rated health (SRH): Odds ratios (95% confidence intervals) across three life stages’ SES strata

| Father’s education | Education | Monthly household income | | | | |
| --- | --- | --- | --- | --- | --- | --- |
|  |  | low |  | medium |  | high |
| **Model 1: crude model** | |  |  |  |  |  |
| illiterate | illiterate | 1.00 |  | 1.31**(1.07-1.59) |  | 1.67**(1.15-2.41) |
|  | primary education | 1.35(0.98-1.86) |  | 1.96***(1.61-2.40) |  | 2.49***(1.83-3.38) |
|  | more than primary | 2.56**(1.32-5.00) |  | 2.77***(1.97-3.90) |  | 3.71***(2.29-6.01) |
| literate | Illiterate | 1.40*(1.01-1.94) |  | 1.41**(1.09-1.81) |  | 3.05***(1.65-5.62) |
|  | Primary education | 1.33(0.92-1.91) |  | 1.97***(1.61-2.41) |  | 2.81***(2.18-3.63) |
|  | more than primary | 2.72**(1.45-5.10) |  | 2.71***(2.17-3.38) |  | 4.10***(3.28-5.12) |
| **Model 2: adjusted for gender and age** | |  |  |  |  |  |
| illiterate | illiterate | 1.00 |  | 1.16(0.95-1.41) |  | 1.39(0.96-2.00) |
|  | primary education | 1.12(0.81-1.53) |  | 1.43***(1.16-1.76) |  | 1.73***(1.27-2.36) |
|  | more than primary | 2.06*(1.08-3.94) |  | 1.94***(1.38-2.75) |  | 2.40***(1.48-3.90) |
| literate | illiterate | 1.49*(1.06-2.09) |  | 1.31*(1.01-1.69) |  | 2.74**(1.49-5.05) |
|  | primary education | 1.13(0.79-1.61) |  | 1.49***(1.21-1.83) |  | 2.05***(1.58-2.67) |
|  | more than primary | 2.06*(1.03-4.10) |  | 1.99***(1.58-2.49) |  | 2.80***(2.22-3.53) |
| **Model 3: adjusted for gender, age and baseline health** | | |  |  |  |  |
| illiterate | illiterate | 1.00 |  | 1.08(0.90-1.28) |  | 1.30(0.94-1.78) |
|  | primary education | 1.20(0.92-1.55) |  | 1.18(0.99-1.42) |  | 1.57**(1.17-2.10) |
|  | more than primary | 1.04(0.59-1.82) |  | 1.70**(1.22-2.38) |  | 1.45(0.92-2.29) |
| literate | illiterate | 1.47*(1.07-2.01) |  | 1.23(0.98-1.55) |  | 2.68***(1.59-4.53) |
|  | primary education | 1.20(0.87-1.64) |  | 1.32**(1.10-1.58) |  | 1.50**(1.18-1.91) |
|  | more than primary | 1.82*(1.01-3.29) |  | 1.52***(1.24-1.87) |  | 1.96***(1.59-2.41) |

* p<0.05, ** p<0.01, *** p<0.001

Table 7B

GEE analysis of ADL disability (bathing only): Odds ratios (95% confidence intervals) across three life stages’ SES strata

| Father’s education | Education | Monthly household income | | | | |
| --- | --- | --- | --- | --- | --- | --- |
|  |  | low |  | medium |  | high |
| **Model 1: crude model** | |  |  |  |  |  |
| illiterate | illiterate | 1.00 |  | 1.47***(1.25-1.73) |  | 1.55*(1.10-2.19) |
|  | primary education | 1.17(0.89-1.53) |  | 1.65***(1.39-1.95) |  | 2.18***(1.58-3.01) |
|  | more than primary | 1.71(0.75-3.91) |  | 1.66**(1.19-2.32) |  | 4.26***(2.10-8.64) |
| literate | illiterate | 1.27(0.93-1.73) |  | 1.41**(1.14-1.74) |  | 1.82*(1.09-3.04) |
|  | primary education | 1.11(0.81-1.52) |  | 1.79***(1.50-2.13) |  | 2.44***(1.83-3.24) |
|  | more than primary | 1.62(0.81-3.22) |  | 1.68***(1.37-2.06) |  | 2.44***(1.96-3.05) |
| **Model 2: adjusted for gender and age** | |  |  |  |  |  |
| illiterate | illiterate | 1.00 |  | 1.20*(1.02-1.41) |  | 1.08(0.78-1.49) |
|  | primary education | 1.15(0.87-1.50) |  | 1.19(0.99-1.42) |  | 1.46*(1.04-2.04) |
|  | more than primary | 1.66(0.79-3.49) |  | 1.22(0.87-1.72) |  | 2.56**(1.33-4.94) |
| literate | illiterate | 1.28(0.93-1.76) |  | 1.12(0.91-1.38) |  | 1.39(0.87-2.23) |
|  | primary education | 1.02(0.74-1.41) |  | 1.29**(1.08-1.55) |  | 1.62***(1.22-2.16) |
|  | more than primary | 1.30(0.69-2.44) |  | 1.27*(1.03-1.57) |  | 1.61***(1.28-2.03) |
| **Model 3: adjusted for gender, age and baseline health** | | |  |  |  |  |
| illiterate | illiterate | 1.00 |  | 1.10(0.94-1.27) |  | 1.04(0.75-1.43) |
|  | primary education | 1.19(0.92-1.54) |  | 1.12(0.94-1.32) |  | 1.44*(1.03-2.00) |
|  | more than primary | 1.74(0.70-4.33) |  | 1.23(0.89-1.70) |  | 2.23*(1.15-4.30) |
| literate | illiterate | 1.26(0.94-1.67) |  | 0.99(0.82-1.21) |  | 1.27(0.76-2.14) |
|  | primary education | 0.97(0.72-1.30) |  | 1.16(0.98-1.38) |  | 1.49**(1.12-1.98) |
|  | more than primary | 1.23(0.64-2.35) |  | 1.16(0.95-1.41) |  | 1.45***(1.16-1.81) |

* p<0.05, ** p<0.01, *** p<0.001

Table 7C

GEE analysis of IADL disability: Odds ratios (95% confidence intervals) across three life stages’ SES strata

| Father’s education | Education | Monthly household income | | | | |
| --- | --- | --- | --- | --- | --- | --- |
|  |  | low |  | medium |  | high |
| **Model 1: crude model** | |  |  |  |  |  |
| illiterate | illiterate | 1.00 |  | 1.88***(1.56-2.27) |  | 2.07***(1.45-2.97) |
|  | primary education | 2.47***(1.85-3.30) |  | 4.51***(3.72-5.47) |  | 6.37***(4.56-8.89) |
|  | more than primary | 2.68*(1.04-6.95) |  | 5.29***(3.72-7.51) |  | 14.26***(6.71-30.31) |
| literate | illiterate | 1.30(0.90-1.88) |  | 2.15***(1.69-2.73) |  | 2.81***(1.58-5.01) |
|  | primary education | 2.79***(1.94-4.02) |  | 4.84***(3.97-5.90) |  | 6.46***(4.89-8.52) |
|  | more than primary | 4.74***(2.34-9.58) |  | 5.30***(4.24-6.61) |  | 8.53***(6.73-10.81) |
| **Model 2: adjusted for gender and age** | |  |  |  |  |  |
| illiterate | illiterate | 1.00 |  | 1.44***(1.20-1.72) |  | 1.32(0.95-1.83) |
|  | primary education | 1.92***(1.44-2.56) |  | 2.47***(2.03-3.01) |  | 3.14***(2.24-4.41) |
|  | more than primary | 1.92(0.77-4.80) |  | 2.75***(1.93-3.92) |  | 6.25***(3.15-12.40) |
| literate | illiterate | 1.40(0.95-2.04) |  | 1.76***(1.39-2.21) |  | 2.18**(1.33-3.56) |
|  | primary education | 2.22***(1.53-3.22) |  | 2.87***(2.35-3.50) |  | 3.49***(2.65-4.60) |
|  | more than primary | 3.04**(1.43-6.45) |  | 3.04***(2.42-3.81) |  | 4.19***(3.30-5.32) |
| **Model 3: adjusted for gender, age and baseline health** | | |  |  |  |  |
| illiterate | illiterate | 1.00 |  | 1.07(0.89-1.28) |  | 1.01(0.67-1.53) |
|  | primary education | 1.20(0.92-1.57) |  | 1.35**(1.10-1.64) |  | 1.60**(1.16-2.20) |
|  | more than primary | 1.65(0.75-3.66) |  | 1.48*(1.06-2.08) |  | 3.08**(1.52-6.26) |
| literate | illiterate | 1.08(0.74-1.59) |  | 1.11(0.88-1.40) |  | 1.50(0.85-2.66) |
|  | primary education | 1.54*(1.09-2.17) |  | 1.48***(1.21-1.80) |  | 1.65***(1.24-2.20) |
|  | more than primary | 1.89*(1.01-3.55) |  | 1.48***(1.17-1.85) |  | 2.06***(1.61-2.63) |

* p<0.05, ** p<0.01, *** p<0.001
