# Supplementary material for: Socioeconomic inequalities in health among elderly people in Taiwan: A life course perspective
Source: PLoS One. 2025 Jul 3;20(7):e0327191. doi: 10.1371/journal.pone.0327191 (PMC12225796; doi:10.1371/journal.pone.0327191)
Supplement: Attachment — Submitted filename: response to reviewers.docx [file pone.0327191.s003.docx]

【section 1】 Editor Comments

1. The authors should explain how categorizing all participants who were "lost to follow-up" as part of the unhealthy group might affect the study's conclusions. Could this approach lead to an overestimation of the association between socioeconomic status and poor health outcomes?

Response:

Thank you for noting that the overestimation of unhealthy data might have resulted from classifying all “lost to follow-up” individuals as unhealthy. We conducted sensitivity analyses to investigate the health outcomes with varying proportions of “lost to follow-up” individuals classified as unhealthy. These analyses were included in the statistical analyses section of the methods on Page# 11, Line 181 of the revised manuscript, with results concluded on Page# 23, Line 259 and the table results detailed in S1 File.

In the sensitivity analyses, we randomly assigned 80% and 90% of individuals “lost to follow-up” as unhealthy, and also tested the outcome when all lost to follow-up individuals were classified as missing. Comparing the sample size of those lost to follow-up with mortality count data from the 2007 TLSA research report, the calculated mortality rate among individuals classified as “lost to follow-up” across five study waves ranged from 83.9% to 92.5%. The comparative data are presented in Table 1 below:

Table 1 Mortality rates among participants classified as “lost to follow-up” across

five waves

| Survey Wave | (A)  Cumulative Sample Size of Those Lost to Follow-up | (B)  Cumulative Mortality Count from 2007 TLSA Research Report | (C)  Number Lost to Follow-up at Each Wave | (D)  Number of Deaths at Each Wave | Dead Proportion at Each Wave, Percentage of (D)/(C) | Cumulative Dead Proportion, Percentage of (B)/(A) |
| --- | --- | --- | --- | --- | --- | --- |
| 1989 | 0 | 0 | 0 | 0 | - | - |
| 1993 | 694 | 582 | 694 | 582 | 83.9% | 83.9% |
| 1996 | 1193 | 1046 | 499 | 464 | 93.0% | 87.7% |
| 1999 | 1642 | 1486 | 449 | 440 | 98.0% | 90.5% |
| 2003 | 2306 | 2133 | 664 | 647 | 97.4% | 92.5% |

In addition, in cohort studies, participants lost to follow-up are rarely lost randomly (Kristman et al., 2004). Studies have found that individuals lost to follow-up tend to belong to lower socioeconomic status groups and experience more health problems, whether these differences are statistically significant or not (Hoeymans et al., 1998; Launer et al., 1994; Mihelic & Crimmins, 1997; Norris, 1985).

The sensitivity analyses yielded similar results to the study. Health outcomes were better for participants with higher socioeconomic status accumulation across three life stages. The magnitude of the results for the 80% and 90% scenarios was attenuated, yet the overall associations and patterns remained stable. The findings from all participants lost to follow-up, categorized as missing, demonstrate an even more pronounced effect on SES accumulation, although they exhibit significant fluctuations and instability. This limitation has been covered in the discussion section; please refer to Page# 28, Line 351 in the revised manuscript.

2. How reliable is it to use a father's education as the only indicator of childhood socioeconomic status? Are there other measures that could provide a more comprehensive understanding of childhood socioeconomic status?

Response:

We are grateful for your reminder to explore other indicators of childhood SES. Common indicators of childhood SES include household income, and the education and occupation of both parents (Bradley & Corwyn, 2002; Kaplan et al., 2001). In the TLSA, data on mothers' socioeconomic status (SES) and childhood household income were not collected, while information on fathers' education and occupation was. By using fathers’ occupation as a measure of childhood SES, we conducted the GEE analysis and found similar results. Please see the results in Table 4-6 at the end of this answer of this question.

Earlier studies often emphasize fathers’ SES, as women's access to education and formal employment has increased only in more recent decades, and childhood household income is prone to recall bias when participants are asked to report it. Therefore, we have selected the father’s education and occupation as the indicators representing childhood SES. Table 2 is the comparative table of sample numbers stratified by fathers’ education and occupation as indicators.

Table 2 Comparative sample sizes stratified by fathers’ education and occupation

| SES indicators | | | N (%) | SES indicators | | | N (%) |
| --- | --- | --- | --- | --- | --- | --- | --- |
| **Father’s**  **education** | Participants'  education | Monthly household income |  | **Father’s**  **occupation** | Participants'  education | Monthly household income |  |
| total |  |  | 4048  (100.0%) | total |  |  | 4048  (100.0%) |
| **illiterate** | illiterate | low | 352(8.7%) | **blue-collar** | illiterate | low | 398(9.8%) |
|  |  | medium | 689(17.0%) |  |  | medium | 819(20.2%) |
|  |  | high | 70(1.7%) |  |  | high | 85(2.1%) |
|  | primary education | low | 118(2.9%) |  | primary education | low | 162(4.0%) |
|  |  | medium | 503(12.4%) |  |  | medium | 764(18.9%) |
|  |  | high | 98(2.4%) |  |  | high | 165(4.1%) |
|  | more than primary | low | 12(0.3%) |  | more than primary | low | 17(0.4%) |
|  |  | medium | 76(1.9%) |  |  | medium | 182(4.5%) |
|  |  | high | 26(0.6%) |  |  | high | 111(2.7%) |
| **literate** | illiterate | low | 82(2.0%) | **white-collar** | illiterate | low | 61(1.5%) |
|  |  | medium | 207(5.1%) |  |  | medium | 141(3.5%) |
|  |  | high | 26(0.6%) |  |  | high | 14(0.3%) |
|  | primary education | low | 72(1.8%) |  | primary education | low | 49(1.2%) |
|  |  | medium | 485(12.0%) |  |  | medium | 310(7.7%) |
|  |  | high | 135(3.3%) |  |  | high | 80(2.0%) |
|  | more than primary | low | 17(0.4%) |  | more than primary | low | 12(0.3%) |
|  |  | medium | 300(7.4%) |  |  | medium | 213(5.3%) |
|  |  | high | 278(6.9%) |  |  | high | 201(5.0%) |
| missing |  |  | 502(12.4%) | missing |  |  | 264(6.5%) |

The data reveal that SES from a preceding stage has a considerable effect on SES in the next stage, with large fluctuations in SES trajectories being rare. Moreover, a higher number of individuals with the most advantaged adulthood SES had literate fathers compared to those with white-collar fathers. The correlation coefficients for each indicator are presented in Table 3. According to Table 3, fathers’ education exhibits a higher correlation with all indicators in comparison to fathers’ occupation. As education serves not only as an indicator of SES but also as a precursor to occupation and income (Liberatos et al., 1988), we used fathers’ education as the proxy for childhood SES instead of occupation.

Table 3 Spearman correlation coefficients for SES indicators and baseline health outcomes

|  | Father’s  occupation | Father’s  education | Participants'  education | Monthly household income | SRH  in 1989 | ADL (bath only)  in 1989 | IADL  in 1989 |
| --- | --- | --- | --- | --- | --- | --- | --- |
| Father’s  occupation | 1.000 |  |  |  |  |  |  |
| Father’s  education | 0.381*** | 1.000 |  |  |  |  |  |
| Participants'  education | 0.332*** | 0.452*** | 1.000 |  |  |  |  |
| Monthly household income | 0.181*** | 0.260*** | 0.357*** | 1.000 |  |  |  |
| SRH  in 1989 | 0.058*** | 0.093*** | 0.199*** | 0.161*** | 1.000 |  |  |
| ADL (bath only)  in 1989 | 0.037* | 0.060*** | 0.093*** | 0.084*** | 0.133*** | 1.000 |  |
| IADL  in 1989 | 0.135*** | 0.209*** | 0.401*** | 0.268*** | 0.245*** | 0.369*** | 1.000 |

* p<0.05, ** p<0.01, *** p<0.001

Table 4

GEE analysis of self-rated health (SRH): odds ratios (95% confidence intervals) across three life stages’ SES strata

| Father’s | Education | Monthly household income | | | | |
| --- | --- | --- | --- | --- | --- | --- |
| occupation |  | low |  | medium |  | high |
| **Model 1: crude model** | |  |  |  |  |  |
| blue-collar | illiterate | 1.00 |  | 1.45***(1.19-1.77) |  | 1.64**(1.13-2.37) |
|  | primary education | 1.36*(1.01-1.83) |  | 2.08***(1.71-2.52) |  | 2.96***(2.32-3.79) |
|  | more than primary | 2.33**(1.26-4.32) |  | 2.84***(2.20-3.66) |  | 4.52***(3.36-6.08) |
| white-collar | illiterate | 1.20(0.81-1.78) |  | 1.22(0.90-1.66) |  | 3.95**(1.62-9.67) |
|  | primary education | 1.57*(1.00-2.46) |  | 2.14***(1.70-2.69) |  | 3.14***(2.26-4.35) |
|  | more than primary | 3.88***(1.95-7.70) |  | 3.34***(2.61-4.27) |  | 4.50***(3.53-5.73) |
| **Model 2: adjusted for gender and age** | |  |  |  |  |  |
| blue-collar | illiterate | 1.00 |  | 1.25*(1.02-1.52) |  | 1.32(0.92-1.90) |
|  | primary education | 1.13(0.84-1.53) |  | 1.46***(1.19-1.79) |  | 2.00***(1.56-2.57) |
|  | more than primary | 1.83*(1.03-3.27) |  | 1.93***(1.48-2.52) |  | 2.83***(2.09-3.83) |
| white-collar | illiterate | 1.27(0.84-1.91) |  | 1.11(0.81-1.50) |  | 3.23*(1.32-7.89) |
|  | primary education | 1.26(0.82-1.93) |  | 1.58***(1.25-2.00) |  | 2.10***(1.50-2.95) |
|  | more than primary | 2.78**(1.29-6.03) |  | 2.41***(1.88-3.09) |  | 2.96***(2.31-3.81) |
| **Model 3: adjusted for gender, age and baseline health** | | |  |  |  |  |
| blue-collar | illiterate | 1.00 |  | 1.18*(1.00-1.40) |  | 1.16(0.85-1.59) |
|  | primary education | 1.23(0.97-1.57) |  | 1.22*(1.03-1.45) |  | 1.60***(1.26-2.02) |
|  | more than primary | 1.39(0.81-2.37) |  | 1.64***(1.29-2.08) |  | 1.97***(1.52-2.56) |
| white-collar | illiterate | 1.49*(1.05-2.13) |  | 1.05(0.80-1.38) |  | 2.87*(1.26-6.52) |
|  | primary education | 1.45(0.98-2.16) |  | 1.50***(1.23-1.83) |  | 1.66***(1.23-2.25) |
|  | more than primary | 1.63(0.87-3.05) |  | 1.80***(1.44-2.25) |  | 1.96***(1.56-2.47) |

* p<0.05, ** p<0.01, *** p<0.001

Table 5

GEE analysis of ADL disability (bathing only): odds ratios (95% confidence intervals) across three life stages’ SES strata

| Father’s | Education | Monthly household income | | | | |
| --- | --- | --- | --- | --- | --- | --- |
| occupation |  | low |  | medium |  | high |
| **Model 1: crude model** | |  |  |  |  |  |
| blue-collar | illiterate | 1.00 |  | 1.51***(1.31-1.75) |  | 1.33(1.00-1.77) |
|  | primary education | 1.11(0.89-1.39) |  | 1.64***(1.42-1.90) |  | 2.05***(1.61-2.61) |
|  | more than primary | 1.19(0.60-2.36) |  | 1.73***(1.38-2.16) |  | 2.35***(1.74-3.17) |
| white-collar | illiterate | 1.19(0.89-1.60) |  | 1.17(0.94-1.45) |  | 2.38*(1.02-5.52) |
|  | primary education | 1.27(0.87-1.87) |  | 1.73***(1.43-2.08) |  | 3.41***(2.39-4.86) |
|  | more than primary | 2.98**(1.36-6.53) |  | 1.73***(1.40-2.15) |  | 2.50***(1.98-3.15) |
| **Model 2: adjusted for gender and age** | |  |  |  |  |  |
| blue-collar | illiterate | 1.00 |  | 1.21**(1.05-1.40) |  | 0.93(0.71-1.23) |
|  | primary education | 1.10(0.89-1.37) |  | 1.21*(1.04-1.40) |  | 1.43**(1.12-1.82) |
|  | more than primary | 1.24(0.67-2.30) |  | 1.31*(1.04-1.65) |  | 1.58**(1.17-2.13) |
| white-collar | illiterate | 1.19(0.89-1.60) |  | 0.96(0.77-1.19) |  | 1.70(0.74-3.92) |
|  | primary education | 1.02(0.71-1.47) |  | 1.23*(1.02-1.49) |  | 2.07***(1.44-2.97) |
|  | more than primary | 1.98(0.89-4.43) |  | 1.34**(1.08-1.66) |  | 1.64***(1.29-2.08) |
| **Model 3: adjusted for gender, age and baseline health** | | |  |  |  |  |
| blue-collar | illiterate | 1.00 |  | 1.12(0.98-1.28) |  | 0.91(0.69-1.20) |
|  | primary education | 1.10(0.89-1.35) |  | 1.09(0.95-1.26) |  | 1.37**(1.08-1.74) |
|  | more than primary | 1.30(0.70-2.44) |  | 1.21(0.96-1.51) |  | 1.48**(1.10-1.98) |
| white-collar | illiterate | 1.14(0.90-1.43) |  | 0.84(0.68-1.03) |  | 1.39(0.61-3.21) |
|  | primary education | 1.02(0.72-1.46) |  | 1.14(0.95-1.37) |  | 1.77**(1.24-2.54) |
|  | more than primary | 1.69(0.76-3.75) |  | 1.22(0.99-1.50) |  | 1.45**(1.16-1.83) |

* p<0.05, ** p<0.01, *** p<0.001

Table 6

GEE analysis of IADL disability: odds ratios (95% confidence intervals) across three life stages’ SES strata

| Father’s | Education | Monthly household income | | | | |
| --- | --- | --- | --- | --- | --- | --- |
| occupation |  | low |  | medium |  | high |
| **Model 1: crude model** | |  |  |  |  |  |
| blue-collar | illiterate | 1.00 |  | 2.08***(1.72-2.50) |  | 2.15***(1.54-3.00) |
|  | primary education | 2.64***(2.03-3.42) |  | 4.76***(3.96-5.72) |  | 6.30***(4.87-8.16) |
|  | more than primary | 2.58*(1.18-5.63) |  | 5.82***(4.53-7.47) |  | 9.34***(6.71-13.00) |
| white-collar | illiterate | 1.03(0.67-1.58) |  | 2.01***(1.54-2.63) |  | 3.46**(1.53-7.84) |
|  | primary education | 2.72***(1.72-4.30) |  | 4.91***(3.93-6.15) |  | 9.39***(6.59-13.38) |
|  | more than primary | 9.63***(4.09-22.70) |  | 5.68***(4.46-7.23) |  | 9.10***(7.03-11.77) |
| **Model 2: adjusted for gender and age** | |  |  |  |  |  |
| blue-collar | illiterate | 1.00 |  | 1.56***(1.30-1.86) |  | 1.38*(1.02-1.87) |
|  | primary education | 2.14***(1.64-2.78) |  | 2.64***(2.20-3.18) |  | 3.30***(2.55-4.27) |
|  | more than primary | 1.96(0.95-4.04) |  | 3.13***(2.41-4.07) |  | 4.29***(3.10-5.95) |
| white-collar | illiterate | 1.11(0.71-1.72) |  | 1.65***(1.27-2.14) |  | 2.30*(1.10-4.83) |
|  | primary education | 1.88**(1.22-2.88) |  | 2.96***(2.37-3.70) |  | 4.60***(3.23-6.55) |
|  | more than primary | 5.55**(1.95-15.85) |  | 3.35***(2.63-4.25) |  | 4.49***(3.49-5.78) |
| **Model 3: adjusted for gender, age and baseline health** | | |  |  |  |  |
| blue-collar | illiterate | 1.00 |  | 1.22*(1.02-1.47) |  | 1.03(0.71-1.49) |
|  | primary education | 1.34*(1.04-1.73) |  | 1.45***(1.20-1.75) |  | 1.70***(1.31-2.21) |
|  | more than primary | 1.70(0.86-3.38) |  | 1.59***(1.23-2.07) |  | 2.26***(1.62-3.17) |
| white-collar | illiterate | 1.14(0.75-1.74) |  | 0.95(0.74-1.21) |  | 1.70(0.62-4.65) |
|  | primary education | 1.30(0.90-1.88) |  | 1.55***(1.24-1.94) |  | 2.18***(1.51-3.15) |
|  | more than primary | 3.14**(1.42-6.95) |  | 1.68***(1.31-2.14) |  | 2.15***(1.65-2.81) |

* p<0.05, ** p<0.01, *** p<0.001

3. How do the observed cohort effects influence the interpretation of the cumulative socioeconomic status on health outcomes? Additionally, how generalizable are these findings to other populations or younger cohorts?

Response:

Thank you for pointing this out. We conducted a GEE analysis by separating participants into two pseudo-cohort groups to observe cohort effects, and found consistent results. Greater accumulation of SES is often linked to superior health outcomes. Please see the results in Table 7-8.

Results for the younger pseudo-cohort remain similar and are reinforced. Conversely, the older pseudo-cohort model is quite unstable. Hence, we conducted another GEE analysis, where all individuals lost to follow-up were classified as missing, as shown in Table 9. The results were akin to those in Table 8. Additionally, Table 10 displays the distribution of the older pseudo-cohort stratified by three SES indicators. It is observed that the survival rate increases with higher SES accumulation. Nevertheless, as reported by the Ministry of the Interior, the average life expectancy in Taiwan was 73.4 years in 1989 and 77.0 years in 2003. Eighty percent of the participant population was lost to follow-up, which might explain the model’s instability.

While we recognize the importance of this consideration, the cohort effect is unavoidable and cannot be analyzed in this manuscript because the dataset lacks sufficient variability. Cumulative effects have been demonstrated in previous studies (Smith et al., 1997), and we observed similar findings in this study, where individuals with more advantaged SES tended to exhibit better health. Despite the fact that contemporary populations generally have higher levels of educational attainment, similar outcomes should be expected, provided that health continues to be shaped by SES. These findings may be generalizable to more advanced studies. This limitation has been addressed in the discussion section, please refer to Page# 27, Line 331 in the revised manuscript.

Table 7

GEE analysis of SRH: odds ratios (95% confidence intervals) across three life stages’ SES strata (participants aged 60-69 years)

| Father’s | Education | Monthly household income | | | | |
| --- | --- | --- | --- | --- | --- | --- |
| education |  | low |  | medium |  | high |
| **Model 1: crude model** | |  |  |  |  |  |
| illiterate | illiterate | 1.00 |  | 1.68**(1.23-2.31) |  | 2.20**(1.34-3.63) |
|  | primary education | 2.14**(1.35-3.40) |  | 2.31***(1.69-3.17) |  | 3.18***(2.14-4.73) |
|  | more than primary | 4.65**(1.84-11.75) |  | 3.71***(2.37-5.81) |  | 4.73***(2.70-8.28) |
| literate | illiterate | 1.67(0.99-2.81) |  | 1.72**(1.18-2.52) |  | 3.66**(1.64-8.19) |
|  | primary education | 1.71(0.99-2.95) |  | 2.43***(1.77-3.34) |  | 3.38***(2.37-4.80) |
|  | more than primary | 2.87**(1.49-5.52) |  | 3.67***(2.64-5.10) |  | 5.08***(3.66-7.05) |
| **Model 2: adjusted for gender and age** | |  |  |  |  |  |
| illiterate | illiterate | 1.00 |  | 1.62**(1.19-2.21) |  | 2.01**(1.22-3.31) |
|  | primary education | 1.91**(1.21-3.02) |  | 1.94***(1.41-2.67) |  | 2.59***(1.74-3.84) |
|  | more than primary | 4.12**(1.60-10.60) |  | 3.07***(1.95-4.84) |  | 3.57***(2.06-6.19) |
| literate | illiterate | 1.80*(1.07-3.03) |  | 1.83**(1.26-2.65) |  | 3.57**(1.65-7.73) |
|  | primary education | 1.55(0.91-2.61) |  | 2.11***(1.54-2.90) |  | 2.87***(2.02-4.10) |
|  | more than primary | 2.50**(1.28-4.86) |  | 3.09***(2.22-4.30) |  | 4.04***(2.90-5.63) |
| **Model 3: adjusted for gender, age and baseline health** | | |  |  |  |  |
| illiterate | illiterate | 1.00 |  | 1.36*(1.06-1.75) |  | 1.55*(1.02-2.34) |
|  | primary education | 1.75**(1.22-2.52) |  | 1.50**(1.17-1.93) |  | 2.16***(1.51-3.08) |
|  | more than primary | 1.70(0.75-3.83) |  | 2.38***(1.58-3.60) |  | 2.01**(1.21-3.33) |
| literate | illiterate | 1.50(0.93-2.40) |  | 1.57**(1.14-2.17) |  | 2.69**(1.40-5.18) |
|  | primary education | 1.54(0.98-2.41) |  | 1.78***(1.38-2.30) |  | 1.89***(1.40-2.56) |
|  | more than primary | 1.94*(1.09-3.47) |  | 2.11***(1.61-2.77) |  | 2.51***(1.91-3.29) |

* p<0.05, ** p<0.01, *** p<0.001

Table 8

GEE analysis of SRH: odds ratios (95% confidence intervals) across three life stages’ SES strata (participants aged 70-96 years)

| Father’s | Education | Monthly household income | | | | |
| --- | --- | --- | --- | --- | --- | --- |
| education |  | low |  | medium |  | high |
| **Model 1: crude model** | |  |  |  |  |  |
| illiterate | illiterate | 1.00 |  | 1.05(0.77-1.42) |  | 0.77(0.31-1.93) |
|  | primary education | 0.78(0.44-1.36) |  | 1.71**(1.23-2.39) |  | 1.30(0.74-2.29) |
|  | more than primary | 1.56(0.57-4.28) |  | 1.39(0.64-3.04) |  | 3.61*(1.00-12.97) |
| literate | illiterate | 1.09(0.65-1.83) |  | 1.26(0.83-1.91) |  | 1.76(0.53-5.90) |
|  | primary education | 1.26(0.73-2.17) |  | 1.58**(1.13-2.21) |  | 2.57***(1.57-4.19) |
|  | more than primary | 3.32(0.60-18.47) |  | 1.80**(1.20-2.69) |  | 3.06***(1.92-4.89) |
| **Model 2: adjusted for gender and age** | |  |  |  |  |  |
| illiterate | illiterate | 1.00 |  | 1.02(0.75-1.37) |  | 0.78(0.32-1.88) |
|  | primary education | 0.70(0.40-1.22) |  | 1.44*(1.01-2.04) |  | 1.21(0.69-2.12) |
|  | more than primary | 1.36(0.51-3.61) |  | 1.09(0.49-2.42) |  | 2.77(0.76-10.66) |
| literate | illiterate | 1.22(0.72-2.07) |  | 1.18(0.78-1.80) |  | 1.66(0.50-5.53) |
|  | primary education | 1.17(0.67-2.03) |  | 1.35(0.96-1.89) |  | 2.15**(1.33-3.48) |
|  | more than primary | 3.34(0.55-20.48) |  | 1.57*(1.04-2.37) |  | 2.62***(1.64-4.17) |
| **Model 3: adjusted for gender, age and baseline health** | | |  |  |  |  |
| illiterate | illiterate | 1.00 |  | 1.04(0.80-1.35) |  | 1.22(0.70-2.13) |
|  | primary education | 0.94(0.63-1.40) |  | 1.05(0.78-1.42) |  | 1.05(0.61-1.81) |
|  | more than primary | 0.79(0.47-1.33) |  | 1.14(0.56-2.33) |  | 1.36(0.50-3.71) |
| literate | illiterate | 1.41(0.90-2.22) |  | 1.22(0.85-1.74) |  | 2.25(0.96-5.28) |
|  | primary education | 1.34(0.79-2.30) |  | 1.07(0.79-1.44) |  | 1.63(0.99-2.67) |
|  | more than primary | 3.85***(1.77-8.40) |  | 1.34(0.91-1.97) |  | 2.10**(1.33-3.33) |

* p<0.05, ** p<0.01, *** p<0.001

Table 9

GEE analysis of SRH: odds ratios (95% confidence intervals) across three life stages’ SES strata (participants aged 70-96 years and all lost to follow-up individuals are excluded)

| Father’s | Education | Monthly household income | | | | |
| --- | --- | --- | --- | --- | --- | --- |
| education |  | low |  | medium |  | high |
| **Model 1: crude model** | |  |  |  |  |  |
| illiterate | illiterate | 1.00 |  | 1.00(0.73-1.36) |  | 0.64(0.27-1.56) |
|  | primary education | 0.79(0.45-1.37) |  | 1.68**(1.19-2.38) |  | 1.29*(1.14-3.81) |
|  | more than primary | 1.93(0.53-7.09) |  | 1.16(0.54-2.46) |  | 4.78*(1.16-19.70) |
| literate | illiterate | 1.20(0.69-2.08) |  | 1.28(0.84-1.96) |  | 1.79(0.52-6.14) |
|  | primary education | 1.36(0.78-2.38) |  | 1.45*(1.03-2.04) |  | 2.48***(1.43-4.30) |
|  | more than primary | 2.14(0.29-15.62) |  | 2.34***(1.51-3.61) |  | 3.18***(1.98-5.10) |
| **Model 2: adjusted for gender and age** | |  |  |  |  |  |
| illiterate | illiterate | 1.00 |  | 0.95(0.69-1.30) |  | 0.66(0.29-1.53) |
|  | primary education | 0.63(0.36-1.11) |  | 1.31(0.91-1.90) |  | 1.70(0.94-3.06) |
|  | more than primary | 1.56(0.46-5.27) |  | 0.90(0.42-1.95) |  | 4.23(0.98-18.21) |
| literate | illiterate | 1.29(0.75-2.24) |  | 1.23(0.81-1.87) |  | 1.91(0.53-6.91) |
|  | primary education | 1.17(0.67-2.03) |  | 1.19(0.84-1.68) |  | 1.97*(1.15-3.38) |
|  | more than primary | 1.81(0.21-15.43) |  | 1.86**(1.19-2.92) |  | 2.64***(1.65-4.22) |
| **Model 3: adjusted for gender, age and baseline health** | | |  |  |  |  |
| illiterate | illiterate | 1.00 |  | 1.00(0.73-1.36) |  | 1.06(0.62-1.81) |
|  | primary education | 0.76(0.47-1.24) |  | 0.93(0.65-1.33) |  | 1.56(0.65-3.75) |
|  | more than primary | 0.83(0.44-1.58) |  | 0.92(0.44-1.91) |  | 1.85(0.73-4.71) |
| literate | illiterate | 1.73*(1.03-2.90) |  | 1.32(0.89-1.96) |  | 2.73*(1.15-6.49) |
|  | primary education | 1.54(0.85-2.80) |  | 0.88(0.61-1.25) |  | 1.79(1.00-3.21) |
|  | more than primary | 2.37(0.75-7.46) |  | 1.83*(1.12-3.01) |  | 2.32***(1.41-3.82) |

* p<0.05, ** p<0.01, *** p<0.001

Table 10 Population size of the older pseudo-cohort stratified by three SES indicators

| SES indicators | | | Total | Remaining | Lost to follow up |
| --- | --- | --- | --- | --- | --- |
| Father’s  education | Participants'  education | Monthly household income |  |  |  |
|  |  |  | N | N(%) | N(%) |
| total |  |  | 1357 | 268(19.7%) | 1089(80.3%) |
| illiterate | illiterate | low | 215 | 37(17.2%) | 178(82.8%) |
|  |  | medium | 269 | 61(22.7%) | 208(77.3) |
|  |  | high | 19 | 6(31.6%) | 13(68.4%) |
|  | primary education | low | 55 | 4(7.3%) | 51(92.7%) |
|  |  | medium | 115 | 28(24.3%) | 87(75.7%) |
|  |  | high | 13 | 1(7.7%) | 12(92.3%) |
|  | more than primary | low | 5 | 1(20.0%) | 4(80.0%) |
|  |  | medium | 16 | 6(37.5%) | 10(62.5%) |
|  |  | high | 4 | 1(25.0%) | 3(75.0%) |
| literate | illiterate | low | 44 | 6(13.6%) | 38(86.4%) |
|  |  | medium | 79 | 15(19.0%) | 64(81.0%) |
|  |  | high | 10 | 2(20.0%) | 8(80.0%) |
|  | primary education | low | 33 | 7(21.2%) | 26(78.8%) |
|  |  | medium | 123 | 31(25.2%) | 92(74.8%) |
|  |  | high | 26 | 8(30.8%) | 18(69.2%) |
|  | more than primary | low | 4 | 1(25.0%) | 3(75.0%) |
|  |  | medium | 72 | 13(18.1%) | 59(81.9%) |
|  |  | high | 43 | 13(30.2%) | 30(69.8%) |
| missing |  |  | 212 | 27(12.7%) | 185(87.3%) |

4. The manuscript contains several grammatical mistakes, specifically on Page# 6, Line 89, Page# 7, Line 114, Page #10, Line 158, and Page# 17, Line 205. I highly recommend conducting a thorough proofread to correct these errors and improve its overall clarity and readability.

Response:

Thank you for your suggestions. We have carefully revised the manuscript to enhance overall clarity and readability, and to correct grammatical errors. Please find the revised manuscript enclosed.

Here are the corrections for the specific points you mentioned:

- Page# 6, Line 89 🡪 Page# 6, Line 103

Origin: “Out of the initial 4,049 participants, only 1,743 remained in the study during the 2003 survey, with 2,306 lost to follow-up.”

Correct: “Of the initial 4,049 participants, 1,743 were retained in the study for the 2003 survey, while 2,306 were lost to follow-up.”

- Page# 7, Line 114 🡪 Page# 8, Line 137

Origin: “Monthly household income levels of respondents were classified into three categories: low, medium, and high.”

Correct: “Respondents' monthly household income levels were categorized into three strata: low (< 3,000 NTD), medium (3,000-19,999 NTD), and high (≥ 20,000 NTD).”

- Page #10, Line 158 🡪 Page# 12, Line 199

Origin: “Generally, populations with more advantageous SES characteristics tend to exhibit better overall health, even after adjusting for demographic factors and baseline health.”

Correct: “Generally, populations characterized by more advantageous SES tend to exhibit better overall health outcomes, even after controlling for demographic factors and baseline health.”

- Page# 17, Line 205 🡪 Page# 22, Line 236

Origin: “Still, superior early and late adulthood SES significantly reflects a lesser decline in health.”

Correct: “Yet, the rate of decline is influenced by SES accumulation, with increased SES accumulation significantly associated with a lesser decline in health.”

【section 2】 Reviewer #1

1- Clarify the specific research questions or hypotheses in the introduction.

Response:

Thank you for pointing this out. The research hypotheses presented in the introduction have been revised as follow. Please also refer to Page# 5, Line 83 in the revised manuscript.

“The purpose of this study is to investigate how health trajectory in old age is shaped by the accumulation of socioeconomic status across three life stages.”

2- would it be possible to Consider exploring potential mediating factors between SES and health outcomes, such as health behaviors or access to healthcare?

Response:

Thank you for highlighting this. While we agree that this is an important consideration, it is beyond the scope of this manuscript. This study examines the impact of SES on health, considering that the small size of subgroup samples could limit further analysis of mediating effects. The correlation coefficients for SES indicators, health behaviors, and baseline health outcomes are presented in Table 11. Table 11 reveals that health behaviors exhibit a mild correlation with all other indicators, which may pose challenges for the analysis of mediating effects.

Table 11 Spearman correlation coefficients for SES indicators, health behaviors, and

baseline health outcomes

|  | Father’s  education | Participants'  education | Monthly household income | Smoke | Drink | SRH  in 1989 | ADL (bath only)  in 1989 | IADL  in 1989 |
| --- | --- | --- | --- | --- | --- | --- | --- | --- |
| Father’s  education | 1.000 |  |  |  |  |  |  |  |
| Participants'  education | 0.452*** | 1.000 |  |  |  |  |  |  |
| Monthly household income | 0.260*** | 0.357*** | 1.000 |  |  |  |  |  |
| Smoke | -0.014 | 0.181*** | 0.056*** | 1.000 |  |  |  |  |
| Drink | 0.083*** | 0.202*** | 0.124*** | 0.314*** | 1.000 |  |  |  |
| SRH  in 1989 | 0.093*** | 0.199*** | 0.161*** | 0.097*** | 0.121*** | 1.000 |  |  |
| ADL (bath only)  in 1989 | 0.060*** | 0.093*** | 0.084*** | 0.080*** | 0.097*** | 0.133*** | 1.000 |  |
| IADL  in 1989 | 0.209*** | 0.401*** | 0.268*** | 0.197*** | 0.211*** | 0.245*** | 0.369*** | 1.000 |

* p<0.05, ** p<0.01, *** p<0.001

As the mediating effect of health behaviors and psychosocial characteristics is well-documented (Lynch et al., 1997; Macintyre, 1997), our study is specifically aimed at examining the direct relationship between SES and health.

3- The study does not address potential reverse causality between SES and health, which could be particularly relevant for late adulthood SES (income) and health outcomes.

Response:

Thank you for highlighting this. While we agree that this is an important consideration, it is beyond the scope of this manuscript due to the need for advanced analytical methods or the inclusion of additional variables.

The effect of health on SES is acknowledged but was not included in our study. The issue of bidirectional causality has been acknowledged since the 1980s, as noted in the Black Report. Table 12 displays the correlation coefficients for SES indicators and baseline health outcomes. While late adulthood SES is significantly related to three health indicators, the effects are weaker than those from early adulthood SES (education), as education represents more than just an SES indicator.

Moreover, most prior research has concentrated on how SES affects health rather than the reverse, as the impact of health on SES is relatively minor compared to the impact of SES on health. We used an early-life health indicator as an example to test the correlation and found similar results. An indicator of early life health is included in Table 12. The health indicator at age 16 was measured in 2003, as it was not surveyed in 1989. Consequently, those lost to follow-up are all classified as unhealthy, potentially leading to an overestimation of early-life unhealthiness. Table 12 reveals the minor yet significant impact of early-life health on SES and health in later life. Our research is limited to examining the effect of SES on health, whilst acknowledging that early-life health can have a minor but profound impact on later-life SES and health (Blane et al., 1993; Haas, 2008; Haas, 2006; West, 1991).

The intricate nature of the SES-health relationship makes it difficult to fully disentangle all the interconnections. Our study focuses on examining the direct relationship between SES and health.

Table 12 Spearman correlation coefficients for SES indicators and health outcomes

|  | Father’s  education | Participants'  education | Monthly household income | SRH  in 1989 | ADL (bath only)  in 1989 | IADL  in 1989 | Health  In 16 years old |
| --- | --- | --- | --- | --- | --- | --- | --- |
| Father’s  education | 1.000 |  |  |  |  |  |  |
| Participants'  education | 0.452*** | 1.000 |  |  |  |  |  |
| Monthly household income | 0.260*** | 0.357*** | 1.000 |  |  |  |  |
| SRH  in 1989 | 0.093*** | 0.199*** | 0.161*** | 1.000 |  |  |  |
| ADL (bath only)  in 1989 | 0.060*** | 0.093*** | 0.084*** | 0.133*** | 1.000 |  |  |
| IADL  in 1989 | 0.209*** | 0.401*** | 0.268*** | 0.245*** | 0.369*** | 1.000 |  |
| Health  In 16 years old | 0.075*** | 0.109*** | 0.126*** | 0.129*** | 0.162*** | 0.203*** | 1.000 |

* p<0.05, ** p<0.01, *** p<0.001

【section 3】 Reviewer #2

**METHODS**

The Methods section is well-structured but could benefit from additional clarity and detail, especially regarding variables and analytical procedures. Suggestions include:

1.1. Describe the study design.

Response:

Thank you for your suggestions. The study design has been added to the revised manuscript; please see Page# 7, Line 117.

“This study aims to explore how cumulative exposure to socioeconomic status (SES) at different life stages shapes health trajectories in later life. SES data were collected in the baseline year, and health trajectories were analyzed using Generalized Estimating Equations (GEE). This design allowed for the examination of baseline health and subsequent changes over time.”

1.2. Detail how the SES variables were categorized and justify these choices. Also, describe how adjusted variables were collected and categorized.

Response:

The three chosen SES indicators are as follows. This study utilized SES indicators across three life stages: childhood, early adulthood, and late adulthood. These indicators included the educational attainment of participants' fathers, the participants' own educational attainment, and monthly household income. The revised version is included on Page# 8, Line 137 of the updated manuscript.

Regarding father’s education, most participants’ fathers from that era had limited access to schooling. This provided an opportunity to examine the health effects of parental education on their offspring, specifically whether the parent received education or not. In the dataset, approximately half of the fathers were literate, while the other half were not.

Educational opportunities improved over time, allowing us to divide participants’ education levels into three categories to capture health disparities between the more advantaged and disadvantaged groups.

A similar rationale was applied to income categorization to enhance the visibility of health differences. The 1989 survey collected data on participants’ monthly household income, categorized as: (1) less than 3,000 NTD, (2) 3,000-4,999 NTD, (3) 5,000-9,999 NTD, (4) 10,000-14,999 NTD, (5) 15,000-19,999 NTD, (6) 20,000-49,999 NTD, and (7) above 50,000 NTD. According to Taiwan’s Ministry of Health and Welfare, the minimum living wage in 1991 was 3,000 NTD. The Directorate-General of Budget, Accounting and Statistics, Executive Yuan, reported an average salary of 21,247 NTD in 1989. Therefore, we used 3,000 NTD and 20,000 NTD as cut points to divide participants into three income groups: low (< 3,000 NTD), medium (3,000-19,999 NTD), and high (≥ 20,000 NTD).

Recognizing the importance of sociodemographic characteristics in determining health, gender and age were included as covariates. The age range of participants was 60 to 96 years. They were classified into five groups, each with a five-year interval starting at age 60, with the last group including those aged 80 and above. This study aims to examine how the accumulation of SES across three life stages informs both baseline health and subsequent health changes. Therefore, baseline health was also controlled for.

1.3. Explain in more detail the ‘hot-deck’ imputation method used for handling missing data. Cite a reference for this technique from the scientific literature as well.

Response:

Missing data on income were imputed using hot-deck imputation, which relies on the modal category based on sex and age. This method is recognized for its computational simplicity and effectiveness, as it leverages values from similar records to provide robust and realistic estimates for the missing data (Cornman et al., 2015; Lee et al., 2015). It has been revised to the updated manuscript; please see Page# 8, Line 139.

1.4. Provide more details about the choice of Generalized Estimating Equations (GEE) models, specifying the type of regression used (Poisson, Logistic, Negative Binomial ...). Also, detail the level of aggregation, wicth probably is the year or the wave.

Response:

Generalized Estimating Equations (GEE) models are particularly well-suited for analyzing data with repeated measures, as they effectively handle correlations within clusters and offer flexibility in specifying the correlation structure. This study employed GEE models with logistic regression, given that the indicators were aggregated into two groups and reported for each wave (Wilson & Lorenz, 2015). It has been revised to the updated manuscript; please see Page# 10, Line 169.

Figure 1 Trend of health indicator performance by year (aggregated)

Figure 1A Self-reported health

Figure 1B ADL (bathing only)

Figure 1C IADL

The GEE results have been refined with superior models; please see Table3-5 on Page# 18-21. Models are evaluated using three correlation structures: Exchangeable, First-order Autoregressive, and Unstructured. The appropriate working correlation structure is selected based on the smallest QIC (Quasi-likelihood under the Independence Model Criterion) value.

1.5. Provide a figure with the model of Analysis Planning for variables organization (DAG - Directed Acyclic Graph).

1.6. Provide more details about the sampling process and the sample size calculation performed.

Response:

Thank you for the reminder. The sampling process is concluded on Page# 6, Line 95 in the revised manuscript.

Sampling process:

A stratified three-stage systematic random sampling method was employed to select 4,412 participants aged 60 years and above. The study population, as of late 1988, resided in 361 administrative units across Taiwan, excluding 30 mountainous areas predominantly inhabited by indigenous populations. The sampling process was conducted in three stages: first, 56 of 331 townships were selected; second, blocks within these townships were sampled; and finally, two elderly individuals (aged ≥60) from each selected block were chosen as survey subjects (*Taiwan Longitudinal Study on Aging (TLSA)*, 2015).

Sample size calculation:

With a population of approximately 1.81 million individuals aged 60 and over from 331 non-mountainous townships and districts, the sampling probability was determined to be 1/410. The age and sex distributions of the sample were then compared to the official household registration data from the end of 1987. In general, the survey results demonstrates substantial representativeness (*Taiwan Longitudinal Study on Aging (TLSA)*, 2015).

**RESULTS**

The Results section does not present the findings clearly and can be improved with more detail and structure.

2.1. Specify the demographic characteristics of the participants in the different waves, as well as response rates and follow-up losses. For example: “Of the initial 4,049 participants, 60% were women, and the average age was 70 years at baseline. In 2003, 1,743 participants remained, with a response rate of approximately 90% in subsequent waves.”

Response:

Thank you for the reminder. The demographic information of the study population has been included in the updated manuscript; please refer to Table 1 on Page# 14.

Of the initial 4,048 participants, 42.9% were women, with an average baseline age of 67.8 years. A total of 48.1% of participants’ fathers were illiterate, 39.8% were literate, and 12.1% lacked relevant data. Approximately 40% of participants were either illiterate or had only received primary education, with less than 20% attaining education more than primary. After implementing Hot-deck imputations, 18.9% of respondents were classified in the low household income group, 63.6% in the medium group, and 17.5% in the high-income group. The response rates for each wave exceeded 91.7%, with attrition rates ranging from 14.9% to 27.6%. By 2003, 1,742 participants remained in the study.

2.2. Provide a flowchart of the sample for each wave.

Response:

Below is the flowchart for each wave; kindly refer to Fig 1 in the revised manuscript as well.

2.3. Create a first table with the sample description stratified by waves (five waves) and include the education variable in the row, as well as the other variables in the study.

Response:

Thank you for the reminder. The demographic information of the study population has been included in the updated manuscript; please refer to Table 1 on Page# 14.

2.4. Tables are not self-explanatory. Reorganize the columns or rows, as it is currently not possible to verify the reference categories in the regressions models.

Response:

Thank you for the reminder. The descriptions of the tables have been revised in the updated manuscript; please refer to Page# 14-21.

For example:

- Table 1, Page# 11 🡪 Table 2, Page# 17

Origin: “Sociodemographic characteristics TLSA in 1989”

Correct: “Characteristics of participants stratified by educational attainment in 1989”

- Table 2, Page# 12 🡪 Table 3, Page# 18

Origin: “GEE analysis for the association of three life stages’ SES indicators with Self-rated health (SRH)”

Correct: “GEE analysis of Self-rated health (SRH): Odds ratios (95% confidence intervals) across three life stages’ SES strata”

**DISCUSSION**

3.1. Avoid being too emphatic in stating that the observed gradients in outcomes are due to cumulative effects, as the study design does not permit this conclusion. Clarify whether this is a cross-sectional or longitudinal cohort study. If the study followed the same individuals from 1989 onwards, it is a cohort study. However, if new individuals were included in each wave, it is cross-sectional, and the waves are time series that can be aggregated using GEE, but these are not cumulative effects, even if parental data were used. Please clarify this point.

Response:

We appreciate the reminder and have thoroughly refined the language to avoid any excessive emphasis; please review the revised manuscript.

This longitudinal cohort study tracked all 4,048 participants from 1989 to 2003, with no new individuals included. Although the TLSA introduced new individuals in later waves, we did not include these new participants in this analysis. Thus, this paper is framed as a cohort study. This has been clarified in the revised manuscript; please refer to Page# 6, Line 91.

3.2. What are the other individual and contextual possible confounding factors not addressed in this study but that might affect the results?

This study's unit of analysis focuses on the individual. While factors at the community and neighborhood (meso) and national and international (macro) levels can also impact individual health (Hertzman et al., 2001), they were not considered in this analysis. For instance, while the sample population was drawn from 331 Taiwanese townships, living standards across townships may vary slightly. Consequently, household income may hold different meanings for different participants, a factor we did not control for. Additionally, government policies and the broader social environment may exert influence on individuals. As literacy has improved over time, educational attainment may carry different implications for different generations (Davey Smith et al., 1998). This cohort effect remains a limitation we could not fully address. These limitations have been addressed in the discussion section; please refer to Page# 26, Line 316.

3.3- Discuss the aging process in more detail.

Response:

Thank you for the reminder. We have addressed this issue in the discussion section to strengthen the argument; please refer to the updated manuscript on Page# 24, Line 275.

From a life course perspective, the development of bodily functions is a dynamic process. Biological systems grow and develop throughout childhood, shaped by early life risk factors. After reaching a plateau at maturity, bodily functions progressively decline with age, with the rate of decline determined by exposures during adulthood. For further information on life course functional trajectories, refer to Figure 2 below and in Power’s 2013 study (Power et al., 2013).

As described above, this study seeks to explore how the level of the maturity plateau, also referred to as baseline health, and the trajectory of subsequent health decline are shaped by SES across three stages of life.

3.4. Similarly to the beginning of the discussion, reconsider the conclusion as it is currently too emphatic and deterministic, which might be biased due to the lack of methodological clarifications still needed.

Response:

We appreciate the reminder and have carefully refined the language to avoid any overly emphatic tone. The clarification has been included in the updated manuscript; please refer to the updated manuscript on Page# 29, Line 371.

“Despite these potential sources of overestimation, our study demonstrates stable gradient health outcomes attributable to the accumulation of SES. Although each subsequent SES exerts its own independent effect on future health, the preceding SES sets the baseline. Policies are better implemented in early life stages to enhance later health and SES. Nevertheless, it is never too late, as each SES at different life stages has its own effect and cannot be offset.”

Reference:

Blane, D., Smith, G. D., & Bartley, M. (1993). Social selection: what does it contribute to social class differences in health? *Sociology of Health & Illness*, *15*(1), 1-15. <https://doi.org/https://doi.org/10.1111/j.1467-9566.1993.tb00328.x>

Bradley, R. H., & Corwyn, R. F. (2002). Socioeconomic Status and Child Development. *Annual Review of Psychology*, *53*(Volume 53, 2002), 371-399. <https://doi.org/https://doi.org/10.1146/annurev.psych.53.100901.135233>

Cornman, J. C., Glei, D. A., Goldman, N., Ryff, C. D., & Weinstein, M. (2015). Socioeconomic Status and Biological Markers of Health:An Examination of Adults in the United States and Taiwan. *Journal of Aging and Health*, *27*(1), 75-102. <https://doi.org/10.1177/0898264314538661>

Davey Smith, G., Hart, C., Hole, D., MacKinnon, P., Gillis, C., Watt, G., Blane, D., & Hawthorne, V. (1998). Education and occupational social class: which is the more important indicator of mortality risk? *Journal of Epidemiology and Community Health*, *52*(3), 153-160. <https://doi.org/10.1136/jech.52.3.153>

Haas, S. (2008). Trajectories of functional health: The ‘long arm’ of childhood health and socioeconomic factors. *Social Science & Medicine*, *66*(4), 849-861. <https://doi.org/https://doi.org/10.1016/j.socscimed.2007.11.004>

Haas, S. A. (2006). Health Selection and the Process of Social Stratification: The Effect of Childhood Health on Socioeconomic Attainment. *Journal of Health and Social Behavior*, *47*(4), 339-354. <https://doi.org/10.1177/002214650604700403>

Hertzman, C., Power, C., Matthews, S., & Manor, O. (2001). Using an interactive framework of society and lifecourse to explain self-rated health in early adulthood. *Soc Sci Med*, *53*(12), 1575-1585. <https://doi.org/10.1016/s0277-9536(00)00437-8>

Hoeymans, N., Feskens, E. J. M., Van Den Bos, G. A. M., & Kromhout, D. (1998). Non-response bias in a study of cardiovascular diseases, functional status and self-rated health among elderly men. *Age and Ageing*, *27*(1), 35-40. <https://doi.org/10.1093/ageing/27.1.35>

Kaplan, G. A., Turrell, G., Lynch, J. W., Everson, S. A., Helkala, E.-L., & Salonen, J. T. (2001). Childhood socioeconomic position and cognitive function in adulthood. *International Journal of Epidemiology*, *30*(2), 256-263. <https://doi.org/10.1093/ije/30.2.256>

Kristman, V., Manno, M., & Côté, P. (2004). Loss to Follow-Up in Cohort Studies: How Much is Too Much? *European Journal of Epidemiology*, *19*(8), 751-760. <https://doi.org/10.1023/B:EJEP.0000036568.02655.f8>

Launer, L. J., Wind, A. W., & Deeg, D. J. H. (1994). Nonresponse Pattern and Bias in a Community-based Cross-sectional Study of Cognitive Functioning among the Elderly. *American Journal of Epidemiology*, *139*(8), 803-812. <https://doi.org/10.1093/oxfordjournals.aje.a117077>

Lee, J., Meijer, E., & Phillips, D. (2015). The effect of using different imputation methods for economic variables in aging surveys. *CESR-Schaeffer Working Paper*(2015-019).

Liberatos, P., Link, B. G., & Kelsey, J. L. (1988). The Measurement of Social Class in Epidemiology. *Epidemiologic Reviews*, *10*(1), 87-121. <https://doi.org/10.1093/oxfordjournals.epirev.a036030>

Lynch, J. W., Kaplan, G. A., & Salonen, J. T. (1997). Why do poor people behave poorly? Variation in adult health behaviours and psychosocial characteristics by stages of the socioeconomic lifecourse. *Social Science & Medicine*, *44*(6), 809-819. <https://doi.org/https://doi.org/10.1016/S0277-9536(96)00191-8>

Macintyre, S. (1997). The black report and beyond what are the issues? *Social Science & Medicine*, *44*(6), 723-745. <https://doi.org/https://doi.org/10.1016/S0277-9536(96)00183-9>

Mihelic, A. H., & Crimmins, E. M. (1997). Loss to Follow-up in a Sample of Americans 70 Years of Age and Older: The LSOA 1984–1990. *The Journals of Gerontology: Series B*, *52B*(1), S37-S48. <https://doi.org/10.1093/geronb/52B.1.S37>

Norris, F. H. (1985). Characteristics of Older Nonrespondents Over Five Waves of a Panel Study1. *Journal of Gerontology*, *40*(5), 627-636. <https://doi.org/10.1093/geronj/40.5.627>

Power, C., Kuh, D., & Morton, S. (2013). From Developmental Origins of Adult Disease to Life Course Research on Adult Disease and Aging: Insights from Birth Cohort Studies. *Annual Review of Public Health*, *34*(Volume 34, 2013), 7-28. <https://doi.org/https://doi.org/10.1146/annurev-publhealth-031912-114423>

Smith, G. D., Hart, C., Blane, D., Gillis, C., & Hawthorne, V. (1997). Lifetime Socioeconomic Position and Mortality: Prospective Observational Study. *BMJ: British Medical Journal*, *314*(7080), 547-552. <http://www.jstor.org/stable/25173845>

*Taiwan Longitudinal Study on Aging (TLSA)*. (2015, 2023/11/08). Health Promotion Administration, Ministry of Health and Welfare, Taiwan. Retrieved 2024/09/06 from <https://www.hpa.gov.tw/Pages/Detail.aspx?nodeid=1077&pid=6197>

Trow, M. (1973). Problems in the transition from elite to mass higher education. *The Carnegie Commission of Higher Education*.

West, P. (1991). Rethinking the health selection explanation for health inequalities. *Social Science & Medicine*, *32*(4), 373-384. <https://doi.org/https://doi.org/10.1016/0277-9536(91)90338-D>

Wilson, J. R., & Lorenz, K. A. (2015). Generalized Estimating Equations Logistic Regression. In J. R. Wilson & K. A. Lorenz (Eds.), *Modeling Binary Correlated Responses using SAS, SPSS and R* (pp. 103-130). Springer International Publishing. <https://doi.org/10.1007/978-3-319-23805-0_6>
