# Supplementary material for: Socioeconomic inequalities in health among elderly people in Taiwan: A life course perspective
Source: PLoS One. 2025 Jul 3;20(7):e0327191. doi: 10.1371/journal.pone.0327191 (PMC12225796; doi:10.1371/journal.pone.0327191)
Supplement: Attachment — Submitted filename: response_to_reviewers_auresp_2.docx [file pone.0327191.s004.docx]

Our heartfelt thanks go to the editor and reviewers. Your thoughtful questions have inspired us to delve deeper into our analysis and improve our explanations.

【section 1】 Editor Comments

- Please review your reference list to ensure that it is complete and correct. If you have cited papers that have been retracted, please include the rationale for doing so in the manuscript text, or remove these references and replace them with relevant current references. Any changes to the reference list should be mentioned in the rebuttal letter that accompanies your revised manuscript. If you need to cite a retracted article, indicate the article’s retracted status in the References list and also include a citation and full reference for the retraction notice.

Response:

Thank you for your reminder, and we apologize for any confusion caused. The references were managed using EndNote, which assigns citation numbers based on the order in the article. Due to the addition, removal, and rearrangement of wording in the three manuscript versions, the citation numbers differ. However, we have meticulously verified that the references are identical across all versions, with only the citation numbers varying. Below is the list of citation numbers for each manuscript version. On this occasion, we opted not to use EndNote for reference management, ensuring there will be no misunderstandings. We appreciate your consideration.

The citation #14 in the original manuscript on Page# 21, Line 266 was mistakenly omitted in the first revised manuscript containing track changes (Page# 31, Line 402). We sincerely apologize for this error.

We have verified all references through PubMed and Google Scholar and have found that no articles have been retracted. Thank you once again for your reminder.

| The numbering in the original manuscript | The numbering in the first revised manuscript with tracked changes | The numbering in the first revised manuscript |
| --- | --- | --- |
| 1-13 | 1-13 | 1-13 |
| - | 14 (new) | 14 |
| 14 | 15 | 15 |
| 15 | 16 | 16 |
| 16 | 17 | 17 |
| - | 18 (new) | 18 |
|  | 19 (new) | 19 |
| 17 | 20 | 20 |
| 18 | 21 | 21 |
| 19 | 22 | 22 |
| - | 23 (new) | 23 |
| 20 | 24 | 24 |
| 21 | 25 | 25 |
| 22 | 26 | 26 |
| 23 | 27 | 27 |
| - | 28 (new) | 28 |
| 24 | 29 | 35 |
| 25 | 30 | 29 |
| 26 | 31 | Delete |
| 27 | 32 | Delete |
| 28 | 33 | 30 |
| 29 | 34 | 31 |
| 30 | 35 | 32 |
| 31 | 36 | 33 |
| 32 | 37 | 34 |
| - | 38 (new) | 36 |
| 33 | 39 | Delete |
| 34 | 40 | Delete |
| - | 41 (new) | 37 |
| - | 42 (new) | 38 |
| - | 43 (new) | 39 |
| 35 | 44 | 40 |
| 36 | 45 | 41 |
| 37 | 46 | 42 |
| 38 | 47 | 43 |
| - | 48 (new) | 44 |
| - | 49 (new) | 45 |
| - | 50 (new) | 46 |
| - | 51 (new) | 47 |
| - | 52 (new) | 48 |

1. Categorizing "lost to follow-up" participants as unhealthy.
   - The authors should extend their sensitivity analysis to include a broader range of scenarios (e.g., 30%, 50%, 70%). This would better demonstrate the robustness of their conclusions across varying assumptions about the health status of those lost to follow-up.
   - The authors could conduct an analysis comparing the SES variables of those lost to follow-up with those who remained in the study. This is an effective way to provide empirical support for the assumption that individuals lost to follow-up tend to have worse SES.

Response:

We appreciate your reminder. Table 1 presents an analysis comparing the SES variables of those lost to follow-up with those who remained in the study across the five waves. The distribution of the three SES indicators for those remaining is significantly different from those lost to follow-up.

We have expanded the sensitivity analysis to include a broader range of scenarios (10%, 30%, 50%, 70%). Please refer to S1 File. Based on the data, 80-90% of those lost to follow-up are deceased, making the GEE results for the 80% and 90% scenarios more aligned with the manuscript results. Given that participants lost to follow-up were randomly classified as unhealthy and the others were assigned to the healthy group, the GEE results for the 10% and 30% scenarios exhibit greater instability, with the 95% confidence intervals being comparably wider across all three health indicators (SRH, ADL, and IADL). As the proportion of individuals lost to follow-up assigned to the unhealthy category increases, the results demonstrate greater stability, and the upper and lower bound values of the 95% confidence intervals show a gradual convergence towards the study's results. Notably, as the percentage scenarios increase, the SES effect becomes more pronounced, which supports the hypothesis of our article. Please also refer to Page# 25 in the revised manuscript.

Table 1

Distribution of three SES indicators for participants remaining and lost to follow-up across five waves

|  | 1989 |  | 1993 | |  | 1996 | |  | 1999 | |  | 2003 | |  |
| --- | --- | --- | --- | --- | --- | --- | --- | --- | --- | --- | --- | --- | --- | --- |
|  | Remaining |  | Remaining | Lost to follow up | X^2^ | Remaining | Lost to follow up | X^2^ | Remaining | Lost to follow up | X^2^ | Remaining | Lost to follow up | X^2^ |
|  | N(%) |  | N(%) | N(%) |  | N(%) | N(%) |  | N(%) | N(%) |  | N(%) | N(%) |  |
| total | 4048  (100.0%) |  | 3354  (100.0%) | 694  (100.0%) |  | 2855  (100.0%) | 1193  (100.0%) |  | 2406  (100.0%) | 1642  (100.0%) |  | 1742  (100.0%) | 2306  (100.0%) |  |
| SES indicators | | | | |  |  |  |  |  |  |  |  |  |  |
| Father’s education | | | | |  |  |  |  |  |  |  |  |  |  |
| illiterate | 1948  (48.1%) |  | 1628  (48.5%) | 320  (46.1%) | 64.78*** | 1379  (48.3%) | 569  (47.7%) | 66.69*** | 1156  (48.0%) | 792  (48.2%) | 48.94*** | 810  (46.5%) | 1138  (49.3%) | 36.87*** |
| literate | 1612  (39.8%) |  | 1383  (41.2%) | 229  (33.0%) |  | 1205  (42.2%) | 407  (34.1%) |  | 1026  (42.6%) | 586  (35.7%) |  | 771  (44.3%) | 841  (36.5%) |  |
| missing | 488  (12.1%) |  | 343  (10.2%) | 145  (20.9%) |  | 271  (9.5%) | 217  (18.2%) |  | 224  (9.3%) | 264  (16.1%) |  | 161  (9.2%) | 327  (14.2%) |  |
| Participants' education | | | | |  |  |  |  |  |  |  |  |  |  |
| illiterate | 1675  (41.4%) |  | 1333  (39.7%) | 342  (49.3%) | 24.19*** | 1104  (38.7%) | 571  (47.9%) | 33.60*** | 909  (37.8%) | 766  (46.7%) | 34.44*** | 625  (35.9%) | 1050  (45.5%) | 43.33*** |
| primary education | 1595  (39.4%) |  | 1366  (40.7%) | 229  (33.0%) |  | 1167  (40.9%) | 428  (35.9%) |  | 990  (41.1%) | 605  (36.8%) |  | 725  (41.6%) | 870  (37.7%) |  |
| more than primary | 760  (18.8%) |  | 642  (19.1%) | 118  (17.0%) |  | 574  (20.1%) | 186  (15.6%) |  | 497  (20.7%) | 263  (16.0%) |  | 383  (22.0%) | 377  (16.3%) |  |
| missing | 18  (0.4%) |  | 13  (0.4%) | 5  (0.7%) |  | 10  (0.4%) | 8  (0.7%) |  | 10  (0.4%) | 8  (0.5%) |  | 9  (0.5%) | 9  (0.4%) |  |
| Monthly household income | | | | |  |  |  |  |  |  |  |  |  |  |
| low | 765  (18.9%) |  | 595  (17.7%) | 170  (24.5%) | 21.70*** | 466  (16.3%) | 299  (25.1%) | 53.04*** | 374  (15.5%) | 391  (23.8%) | 55.35*** | 248  (14.2%) | 517  (22.4%) | 63.84*** |
| medium | 2575  (63.6%) |  | 2145  (64.0%) | 430  (62.0%) |  | 1838  (64.4%) | 737  (61.8%) |  | 1554  (64.6%) | 1021  (62.2%) |  | 1119  (64.2%) | 1456  (63.1%) |  |
| high | 708  (17.5%) |  | 614  (18.3%) | 94  (13.5%) |  | 551  (19.3%) | 157  (13.2%) |  | 478  (19.9%) | 230  (14.0%) |  | 375  (21.5%) | 333  (14.4%) |  |

* p<0.05, ** p<0.01, *** p<0.001

2. Cohort effects and generalizability of findings.

- Response: The authors addressed cohort effects through analyses of pseudo-cohorts and discussed the limitations of dataset variability.
- Recommend that the authors include age as a time-varying covariate in their Generalized Estimating Equations (GEE) model. This would allow them to better differentiate between the effects of biological aging and cohort effects.

Response:

Thank you for your suggestion. The limitation regarding the generational issue has been included in the revised manuscript. Please refer to Page# 30, Line 409.

Age was employed as a time-varying covariate to manage the effects of biological ageing, with age being controlled across all five waves. Meanwhile, the five age groups were treated as a control factor for cohort effects instead of for ageing issue. The GEE results are presented in Tables 2-4 below.

The results in Tables 2-4 indicate that health outcomes continue to be influenced by SES. However, while the gradient persists, different health indicators exhibit varying degrees of influence. The influence of SES on IADL remains notably significant, whereas effects on ADL have largely dissipated. Only the most advantaged SES populations still demonstrate significantly better ADL outcomes compared to the most disadvantaged group. Even though most ADL results are not stable after adjusting for age across five waves (biological ageing) and age group (cohort effect), a gradient is still observed. With higher levels of SES, the odds ratios and 95% confidence intervals increase correspondingly.

Thank you for highlighting this issue. From a life course perspective, growth occurs during childhood, shaped by early risk factors. After reaching a plateau at maturity, bodily functions progressively decline with age, with the rate of decline determined by exposures during adulthood (Power et al., 2013). Based on the theory, our study focuses on health shaped by the accumulation of SES, which includes baseline health and the ageing process during the survey years. When controlling for age across five waves, we can only observe the period effect, not the ageing effect. Therefore, we tend not to control for age across the five waves. When we control for gender, baseline age, and baseline health in the model 3, it allows us to observe the rate of decline to ascertain whether the ageing process is influenced by SES. Thank you for giving us the opportunity to clarify our analysis. This issue is discussed in more detail in the updated manuscript on Page# 26, Line 339.

Table 2

GEE analysis of self-rated health (SRH): odds ratios (95% confidence intervals) across three life stages’ SES strata

| Father’s | Education | Monthly household income | | | | |
| --- | --- | --- | --- | --- | --- | --- |
| education |  | low |  | medium |  | high |
| **Model 1: crude model** | |  |  |  |  |  |
| illiterate | illiterate | 1.00 |  | 1.43**(1.15-1.77) |  | 1.76**(1.17-2.67) |
|  | primary education | 1.46*(1.03-2.07) |  | 2.17***(1.75-2.70) |  | 2.88***(2.10-3.95) |
|  | more than primary | 3.11**(1.51-6.42) |  | 3.12***(2.17-4.50) |  | 4.55***(2.80-7.39) |
| literate | illiterate | 1.35(0.94-1.95) |  | 1.55**(1.18-2.03) |  | 2.82**(1.46-5.46) |
|  | primary education | 1.50*(1.02-2.21) |  | 2.21***(1.78-2.75) |  | 3.22***(2.48-4.17) |
|  | more than primary | 2.95***(1.61-5.41) |  | 3.17***(2.51-4.00) |  | 4.73***(3.73-5.98) |
| **Model 2: adjusted for gender, age across five waves and age group** | | |  |  |  |  |
| illiterate | illiterate | 1.00 |  | 1.22(0.98-1.53) |  | 1.40(0.91-2.14) |
|  | primary education | 1.17(0.82-1.67) |  | 1.52***(1.20-1.92) |  | 1.90***(1.36-2.66) |
|  | more than primary | 2.81**(1.35-5.82) |  | 2.11***(1.42-3.13) |  | 2.92***(1.73-4.92) |
| literate | illiterate | 1.49*(1.01-2.19) |  | 1.36*(1.02-1.81) |  | 2.40*(1.19-4.85) |
|  | primary education | 1.24(0.82-1.86) |  | 1.58***(1.25-2.00) |  | 2.19***(1.65-2.92) |
|  | more than primary | 2.11*(1.03-4.33) |  | 2.27***(1.77-2.92) |  | 3.18***(2.46-4.12) |
| **Model 3: adjusted for gender, age across five waves, age group and baseline health** | | | | |  |  |
| illiterate | illiterate | 1.00 |  | 1.14(0.94-1.37) |  | 1.30(0.90-1.86) |
|  | primary education | 1.28(0.96-1.70) |  | 1.21(1.00-1.47) |  | 1.73**(1.25-2.40) |
|  | more than primary | 1.22(0.66-2.27) |  | 1.85**(1.28-2.68) |  | 1.62(1.00-2.63) |
| literate | illiterate | 1.46*(1.03-2.07) |  | 1.27(0.99-1.64) |  | 2.38**(1.33-4.24) |
|  | primary education | 1.40(0.98-2.02) |  | 1.39**(1.14-1.69) |  | 1.52**(1.18-1.97) |
|  | more than primary | 1.88*(1.01-3.48) |  | 1.69***(1.35-2.11) |  | 2.13***(1.70-2.67) |

* p<0.05, ** p<0.01, *** p<0.001

Table 3

GEE analysis of ADL disability (bathing only): odds ratios (95% confidence intervals) across three life stages’ SES strata

| Father’s | Education | Monthly household income | | | | |
| --- | --- | --- | --- | --- | --- | --- |
| education |  | low |  | medium |  | high |
| **Model 1: crude model** | |  |  |  |  |  |
| illiterate | illiterate | 1.00 |  | 1.44***(1.24-1.68) |  | 1.50*(1.08-2.07) |
|  | primary education | 1.13(0.87-1.45) |  | 1.60***(1.36-1.88) |  | 2.16***(1.58-2.94) |
|  | more than primary | 1.70(0.69-4.18) |  | 1.72**(1.24-2.38) |  | 4.30***(2.22-8.34) |
| literate | illiterate | 1.18(0.88-1.58) |  | 1.40**(1.14-1.71) |  | 1.65(0.98-2.78) |
|  | primary education | 1.13(0.83-1.54) |  | 1.74***(1.47-2.05) |  | 2.48***(1.89-3.24) |
|  | more than primary | 1.65(0.87-3.14) |  | 1.69***(1.39-2.05) |  | 2.40***(1.94-2.95) |
| **Model 2: adjusted for gender, age across five waves and age group** | | |  |  |  |  |
| illiterate | illiterate | 1.00 |  | 1.20(0.96-1.49) |  | 1.08(0.69-1.69) |
|  | primary education | 1.19(0.83-1.72) |  | 1.15(0.90-1.47) |  | 1.62*(1.06-2.49) |
|  | more than primary | 2.26(0.75-6.79) |  | 1.40(0.88-2.21) |  | 3.29**(1.53-7.11) |
| literate | illiterate | 1.51*(1.00-2.26) |  | 1.07(0.79-1.43) |  | 1.23(0.61-2.50) |
|  | primary education | 0.98(0.62-1.53) |  | 1.31*(1.02-1.67) |  | 1.74**(1.19-2.54) |
|  | more than primary | 1.43(0.59-3.47) |  | 1.35*(1.02-1.80) |  | 1.78***(1.31-2.41) |
| **Model 3: adjusted for gender, age across five waves, age group and baseline health** | | | | |  |  |
| illiterate | illiterate | 1.00 |  | 1.08(0.86-1.34) |  | 1.01(0.63-1.61) |
|  | primary education | 1.22(0.84-1.78) |  | 1.05(0.82-1.35) |  | 1.57*(1.02-2.42) |
|  | more than primary | 2.47(0.78-7.78) |  | 1.39(0.87-2.24) |  | 2.96**(1.35-6.49) |
| literate | illiterate | 1.47(0.99-2.18) |  | 0.92(0.68-1.24) |  | 1.12(0.54-2.34) |
|  | primary education | 0.94(0.59-1.48) |  | 1.17(0.91-1.49) |  | 1.58*(1.07-2.32) |
|  | more than primary | 1.45(0.59-3.58) |  | 1.23(0.92-2.20) |  | 1.62**(1.19-2.20) |

* p<0.05, ** p<0.01, *** p<0.001

Table 4

GEE analysis of IADL disability: odds ratios (95% confidence intervals) across three life stages’ SES strata

| Father’s | Education | Monthly household income | | | | |
| --- | --- | --- | --- | --- | --- | --- |
| education |  | low |  | medium |  | high |
| **Model 1: crude model** | |  |  |  |  |  |
| illiterate | illiterate | 1.00 |  | 2.05***(1.68-2.50) |  | 2.27***(1.58-3.26) |
|  | primary education | 2.65***(1.96-3.59) |  | 4.92***(4.01-6.03) |  | 6.82***(4.92-9.45) |
|  | more than primary | 3.15*(1.18-8.42) |  | 6.05***(4.27-8.58) |  | 16.24***(7.93-33.29) |
| literate | illiterate | 1.36(0.93-2.01) |  | 2.35***(1.83-3.03) |  | 3.16***(1.75-5.70) |
|  | primary education | 2.85***(1.96-4.12) |  | 5.21***(4.23-6.41) |  | 7.55***(5.70-9.99) |
|  | more than primary | 5.77***(2.86-11.67) |  | 5.87***(4.68-7.37) |  | 9.32***(7.31-11.87) |
| **Model 2: adjusted for gender, age across five waves and age group** | | |  |  |  |  |
| illiterate | illiterate | 1.00 |  | 1.63***(1.30-2.03) |  | 1.46(0.97-2.18) |
|  | primary education | 2.32***(1.63-3.31) |  | 3.05***(2.40-3.88) |  | 3.98***(2.69-5.89) |
|  | more than primary | 2.73(0.91-8.22) |  | 3.76***(2.47-5.71) |  | 9.48***(4.53-19.84) |
| literate | illiterate | 1.67*(1.07-2.61) |  | 2.06***(1.55-2.73) |  | 2.67**(1.46-4.90) |
|  | primary education | 2.63***(1.68-4.13) |  | 3.60***(2.83-4.58) |  | 4.66***(3.35-6.50) |
|  | more than primary | 4.66***(1.91-11.39) |  | 3.96***(3.02-5.21) |  | 5.59***(4.20-7.44) |
| **Model 3: adjusted for gender, age across five waves, age group and baseline health** | | | | |  |  |
| illiterate | illiterate | 1.00 |  | 1.10(0.88-1.37) |  | 1.05(0.64-1.73) |
|  | primary education | 1.26(0.91-1.74) |  | 1.39**(1.10-1.76) |  | 1.77**(1.23-2.57) |
|  | more than primary | 2.32(0.88-6.13) |  | 1.74**(1.16-2.62) |  | 4.16***(1.86-9.27) |
| literate | illiterate | 1.22(0.80-1.87) |  | 1.11(0.84-1.48) |  | 1.63(0.84-3.15) |
|  | primary education | 1.68*(1.10-2.55) |  | 1.56***(1.23-1.98) |  | 1.78***(1.27-2.49) |
|  | more than primary | 2.41*(1.10-5.24) |  | 1.64***(1.25-2.14) |  | 2.31***(1.74-3.08) |

* p<0.05, ** p<0.01, *** p<0.001

【section 2】 Reviewer #2

1- Study design and methodological clarifications.
   It could be improved with more detail and clarity about the study’s design and methodology such as whether this is a cohort study or explain key design elements, such as the timeline, follow-up intervals and a brief description of how participants were recruited and followed over time.

Response:

Thank you for the reminder. Additional details about the TLSA have been incorporated into the revised manuscript. Kindly refer to Page# 6, Section “Study Data and Design”.

2- Detail about SES variables:
   Why did not authors use methods like Principal Component Analysis (PCA) to extract a single composite SES variable?

Response:

Thank you for highlighting this. Given the robust effect of SES on health and the profound influence of the childhood SES on later health, this study observed the phenomenon once again. The study design helps to illustrate the intertwined relationship, which may not be captured if using PCA for analysis.

The data indicate that the effects of each SES indicator are independent. Furthermore, previous studies that classify those subgroups of populations with the same SES indicator as having uniform health conditions overlook the fact that their health status varies due to different SES levels experienced throughout their life trajectories. Each stage of life leaves its mark on the individual. Disadvantaged SES imprints on individuals, even if they later achieve better SES. For instance, those with the highest income but lower educational attainment exhibit slightly worse health, a status not offset by their later advantaged SES. As a result, it is essential to acknowledge that these subgroups are not regarded as having the same health status.

Nonetheless, the health statuses of these subgroups are not purely independently different. Given that these subgroups share the same SES indicators with two other different SES, their health statuses are relevant to one another.

When using PCA for analysis, we might not detect distinctions among these subgroups. We would treat subgroups under the same SES indicator as entirely different groups, since PCA provides only a composite score to evaluate all SES indicators together. We greatly appreciate your suggestion of this method. In clarifying why PCA was not chosen, we can articulate our study design more clearly. This issue is discussed in the updated manuscript on Page# 27, Line 344.

3- Results.
   I could not find any changes regarding comment 2.1 in the manuscript, or I may be missing something.

Response:

Thank you for the reminder. In our first revised manuscript, we aimed to simplify the article and focus on the main points, as the original manuscript contained too much information, leading to confusion. Thus, we presented the demographic characteristics only in Table 1. We have now included the description in the text. Kindly refer to Page# 13, Line 216.

Reference:

Power, C., Kuh, D., & Morton, S. (2013). From Developmental Origins of Adult Disease to Life Course Research on Adult Disease and Aging: Insights from Birth Cohort Studies. *Annual Review of Public Health*, *34*(Volume 34, 2013), 7-28. <https://doi.org/https://doi.org/10.1146/annurev-publhealth-031912-114423>
