# Supplementary material for: Socioeconomic inequalities in health among elderly people in Taiwan: A life course perspective
Source: PLoS One. 2025 Jul 3;20(7):e0327191. doi: 10.1371/journal.pone.0327191 (PMC12225796; doi:10.1371/journal.pone.0327191)
Supplement: Attachment — Submitted filename: response_to_reviewers_auresp_3.docx [file pone.0327191.s005.docx]

【Review Comments】

- Does the study design sufficiently account for potential biases introduced by the loss of follow-up participants? It may benefit from a more direct explanation of how these objectives specifically relate to the study's aims regarding socioeconomic status (SES) and health trajectories.

Response:

We appreciate your reminder. The constraints inherent in this study restricted the ability to achieve a fully robust design. Given that the majority of participants lost to follow-up were deceased, they were categorized as unhealthy to better analyze the association between socioeconomic status (SES) and health trajectories. Comprehensive sensitivity analyses were conducted to ensure the stability and reliability of the data.

Comparing the sample size of those lost to follow-up with mortality count data from the 2007 TLSA research report, the calculated mortality rate among individuals classified as “lost to follow-up” across five study waves ranged from 83.9% to 92.5%. The comparative data are provided in Table 1 for further reference.

Table 1 Mortality rates among participants classified as “lost to follow-up” across

five waves

| Survey Wave | (A)  Cumulative Sample Size of Those Lost to Follow-up | (B)  Cumulative Mortality Count from 2007 TLSA Research Report | (C)  Number Lost to Follow-up at Each Wave | (D)  Number of Deaths at Each Wave | Dead Proportion at Each Wave, Percentage of (D)/(C) | Cumulative Dead Proportion, Percentage of (B)/(A) |
| --- | --- | --- | --- | --- | --- | --- |
| 1989 | 0 | 0 | 0 | 0 | - | - |
| 1993 | 694 | 582 | 694 | 582 | 83.9% | 83.9% |
| 1996 | 1193 | 1046 | 499 | 464 | 93.0% | 87.7% |
| 1999 | 1642 | 1486 | 449 | 440 | 98.0% | 90.5% |
| 2003 | 2306 | 2133 | 664 | 647 | 97.4% | 92.5% |

- The exclusion of mountainous areas merits more justification. How might this exclusion impact the generalizability of the findings?

Response:

We appreciate your reminder. The relationship between SES and health is firmly established. Moreover, the multifaceted interactions both within various SES dimensions and between SES and health exhibit considerable complexity. This study aimed to further explore this relationship by elucidating the connections between SES at three distinct life stages and later health trajectories. The health gradients were examined from multiple perspectives. An interesting observation emerged, which may merit further exploration. These findings may be generalizable to more advanced studies.

- Lines 127-129: Please provide more details regarding the reasons for participant attrition. Were there common factors among those lost to follow-up that could introduce selection bias?

Response:

Thank you for pointing this out. As shown in Table 1, the majority of participants lost to follow-up were deceased, while the remaining individuals either declined participation, were repeatedly untraceable, or lost contact entirely. Nonetheless, in each wave, only approximately 10% of the remaining participants were not included in the investigation. This survey was conducted under the coordination of the Health Promotion Administration, Ministry of Health and Welfare, and benefited significantly from local authorities' assistance. The reliability of the data was ensured despite certain losses.

However, in cohort studies, participants lost to follow-up are rarely lost randomly (Kristman et al., 2004). Studies have found that individuals lost to follow-up tend to belong to lower socioeconomic status groups and experience more health problems, whether these differences are statistically significant or not (Hoeymans et al., 1998; Launer et al., 1994; Mihelic & Crimmins, 1997; Norris, 1985).

The remaining participants generally have higher SES, potentially leading to selection bias. While we acknowledge the significance of this factor, the loss of participants was inevitable. Despite the fact, similar outcomes should be expected, provided that health continues to be shaped by SES.

- Lines 160-167: Consider adding context about the significance of the chosen income categories. How do these thresholds correlate with the economic context of Taiwan during the relevant years?

Response:

The 1989 survey collected data on participants’ monthly household income, categorized as: (1) less than 3,000 NTD, (2) 3,000-4,999 NTD, (3) 5,000-9,999 NTD, (4) 10,000-14,999 NTD, (5) 15,000-19,999 NTD, (6) 20,000-49,999 NTD, and (7) above 50,000 NTD. According to Taiwan’s Ministry of Health and Welfare, the minimum living wage in 1991 was 3,000 NTD. The Directorate-General of Budget, Accounting and Statistics, Executive Yuan, reported an average salary of 21,247 NTD in 1989. Therefore, we used 3,000 NTD and 20,000 NTD as cut points to divide participants into three income groups: low (< 3,000 NTD), medium (3,000-19,999 NTD), and high (≥ 20,000 NTD).

- Lines 172-185: While the selection of health indicators appears rational, the focus on bathing as an Activities of Daily Living (ADL) measure seems somewhat narrow. Please justify the choice of bathing over other potential indicators, such as eating or dressing.

Response:

The ADLs include the following: “(1) Ambulating: The Extent of an individual's ability to move from 1 position to another and walk independently; (2) Feeding: The Ability of an individual to feed oneself; (3) Dressing: The ability to select appropriate clothes and to put them on; (4) Personal hygiene: The ability to bathe and groom oneself and maintain dental hygiene, nail, and hair care; (5) Continence: The ability to control bladder and bowel function; (6) Toileting: The ability to get to and from the toilet, use it appropriately, and clean oneself afterward” (Edemekong et al., 2025).

In the 1989 interview, participants were asked about their ability to: (1) independently purchase daily necessities, such as soap, toothpaste, and medicine; (2) manage financial matters, including paying bills and handling change; (3) make phone calls; (4) maintain personal hygiene, specifically through bathing; (5) climb stairs; (6) walk a distance of 200–300 meters; (7) perform labor-intensive tasks, such as floor cleaning and agricultural work; (8) manage transportation, either by driving or arranging alternative modes; (9) lift objects weighing approximately 12 kg; (10) perform squatting movements; (11) raise arms above head level; (12) manipulate objects using fine finger movements; and (13) maintaining a standing position for a continuous duration of two hours.

Due to the incomplete ADL data in the 1989 interview, only bathing was selected as the representative ADL indicator for this study. Nevertheless, in Taiwan, difficulty bathing is the most frequently cited limitation in ADL. Having persistent difficulties bathing is an independent risk of long-term nursing home admission (Gill et al., 2006).

- The abbreviations for TLSA, SES, and others were not introduced in Figure 1 and Tables. Please clarify these terms for better understanding.

Response:

We appreciate your reminder. Kindly review the revised Figure 1, the updated Table 1, and the supporting information.

- Regarding the Self-Rated Health (SRH) question with a 5-point Likert scale, which specific points were considered indicative of "good" health?

Response:

Thank you for pointing this out. For the purposes of this study, we designated "very good" and "good" health as "good," whereas the other three classifications were categorized as "poor" health, given our analytical focus on overall health status.

Reference:

Edemekong PF, Bomgaars DL, Sukumaran S, et al. Activities of Daily Living. [Updated 2025 May 4]. In: StatPearls [Internet]. Treasure Island (FL): StatPearls Publishing; 2025 Jan-. Available from: <https://www.ncbi.nlm.nih.gov/books/NBK470404/>

Gill TM, Allore HG, Han L. Bathing disability and the risk of long-term admission to a nursing home. J Gerontol A Biol Sci Med Sci. 2006;61(8):821-5.

Hoeymans, N., Feskens, E. J. M., Van Den Bos, G. A. M., & Kromhout, D. (1998). Non-response bias in a study of cardiovascular diseases, functional status and self-rated health among elderly men. *Age and Ageing*, *27*(1), 35-40. <https://doi.org/10.1093/ageing/27.1.35>

Kristman, V., Manno, M., & Côté, P. (2004). Loss to Follow-Up in Cohort Studies: How Much is Too Much? *European Journal of Epidemiology*, *19*(8), 751-760. <https://doi.org/10.1023/B:EJEP.0000036568.02655.f8>

Launer, L. J., Wind, A. W., & Deeg, D. J. H. (1994). Nonresponse Pattern and Bias in a Community-based Cross-sectional Study of Cognitive Functioning among the Elderly. *American Journal of Epidemiology*, *139*(8), 803-812. <https://doi.org/10.1093/oxfordjournals.aje.a117077>

Mihelic, A. H., & Crimmins, E. M. (1997). Loss to Follow-up in a Sample of Americans 70 Years of Age and Older: The LSOA 1984–1990. *The Journals of Gerontology: Series B*, *52B*(1), S37-S48. <https://doi.org/10.1093/geronb/52B.1.S37>

Norris, F. H. (1985). Characteristics of Older Nonrespondents Over Five Waves of a Panel Study1. *Journal of Gerontology*, *40*(5), 627-636. <https://doi.org/10.1093/geronj/40.5.627>
